# Expression and regulation of type 2A protein phosphatases and alpha4 signalling in cardiac health and hypertrophy

**DOI:** 10.1007/s00395-017-0625-2

**Published:** 2017-05-19

**Authors:** Olga Eleftheriadou, Andrii Boguslavskyi, Michael R. Longman, Jonathan Cowan, Asvi Francois, Richard J. Heads, Brian E. Wadzinski, Ali Ryan, Michael J. Shattock, Andrew K. Snabaitis

**Affiliations:** 1 School of Life Sciences, Pharmacy and Chemistry, Faculty of Science Engineering and Computing, Kingston University, Penrhyn Road, Kingston-upon-Thames, Surrey, KT1 2EE UK; 2 Cardiovascular Division, King’s College London British Heart Foundation Centre, The Rayne Institute, St Thomas’ Hospital, London, SE1 7EH UK; 3 Department of Pharmacology, Vanderbilt University, Nashville, TN USA

**Keywords:** Type 2A protein phosphatase, Alpha4, Cardiac hypertrophy, Hydrogen peroxide, H2A.X

## Abstract

Cardiac physiology and hypertrophy are regulated by the phosphorylation status of many proteins, which is partly controlled by a poorly defined type 2A protein phosphatase-alpha4 intracellular signalling axis. Quantitative PCR analysis revealed that mRNA levels of the type 2A catalytic subunits were differentially expressed in H9c2 cardiomyocytes (PP2ACβ > PP2ACα > PP4C > PP6C), NRVM (PP2ACβ > PP2ACα = PP4C = PP6C), and adult rat ventricular myocytes (PP2ACα > PP2ACβ > PP6C > PP4C). Western analysis confirmed that all type 2A catalytic subunits were expressed in H9c2 cardiomyocytes; however, PP4C protein was absent in adult myocytes and only detectable following 26S proteasome inhibition. Short-term knockdown of alpha4 protein expression attenuated expression of all type 2A catalytic subunits. Pressure overload-induced left ventricular (LV) hypertrophy was associated with an increase in both PP2AC and alpha4 protein expression. Although PP6C expression was unchanged, expression of PP6C regulatory subunits (1) Sit4-associated protein 1 (SAP1) and (2) ankyrin repeat domain (ANKRD) 28 and 44 proteins was elevated, whereas SAP2 expression was reduced in hypertrophied LV tissue. Co-immunoprecipitation studies demonstrated that the interaction between alpha4 and PP2AC or PP6C subunits was either unchanged or reduced in hypertrophied LV tissue, respectively. Phosphorylation status of phospholemman (Ser63 and Ser68) was significantly increased by knockdown of PP2ACα, PP2ACβ, or PP4C protein expression. DNA damage assessed by histone H2A.X phosphorylation (γH2A.X) in hypertrophied tissue remained unchanged. However, exposure of cardiomyocytes to H_2_O_2_ increased levels of γH2A.X which was unaffected by knockdown of PP6C expression, but was abolished by the short-term knockdown of alpha4 expression. This study illustrates the significance and altered activity of the type 2A protein phosphatase-alpha4 complex in healthy and hypertrophied myocardium.

**Electronic supplementary material:**

The online version of this article (doi:10.1007/s00395-017-0625-2) contains supplementary material, which is available to authorized users.

## Introduction

Serine/threonine protein phosphatases and kinases control the phosphorylation status of many substrates and, as a consequence, regulate their activity and/or cellular localisation. The serine/threonine protein phosphatases comprise a relatively large family of enzymes that are ubiquitously expressed and can be further subdivided into PP1, PP2A, PP2B (calcineurin), PP2C, PP3, PP5, and PP7 families based on their sensitivity to inhibitors such as okadaic acid [15] and their metal ion-dependent activity [14, 33]. The type 2A protein phosphatase (PP2A) family, named after the first member of this family (PP2A) to be cloned [2], was later found to be comprised of highly homologous PP4 (PPX) and PP6 (PPV) catalytic subunit proteins [15]. The catalytic subunits (C) are responsible for conferring phosphatase enzyme activity towards a plethora of substrates. However, despite the high sequence homology between the type 2A phosphatase catalytic subunits (>50%), there is very little similarity between the mechanisms that control their cellular activity and localisation.

To avoid indiscriminate phosphatase activity, PP2AC subunits do not exist as “free” monomers in the cytosol, but are usually associated with other regulatory proteins and consequently, PP2AC can exist in several discreet heteromeric populations. In its simplest form, the catalytic subunit associates with a scaffold (A) subunit to form a heterodimeric core enzyme [43, 68] that constitutes approximately a third of total PP2A in the cell [43]. Furthermore, the scaffold A- and C-subunits are expressed in varying degrees as α and β isoforms [3, 27, 30]; however, the complexity of activity towards substrates is largely controlled by the association of the heterodimeric core enzyme with a non-catalytic regulatory/targeting B subunit to form the active heterotrimeric holoenzyme [36]. The B subunits are themselves subdivided into four distinct gene families that give rise to the B, B′, B″, and B″′ subfamilies [36] encoded by 15 different human genes giving rise to at least 23 different isoforms (reviewed by Sents et al. [72]). The architecture of the heterotrimeric PP2A complex can result in at least 96 distinct holoenzymes [37].

It is clear that the activity and localisation of type 2A protein phosphatase catalytic subunits are regulated by their association with a myriad of accessory proteins. One such atypical regulatory subunit for the type 2A phosphatases, namely alpha4, was first cloned from B-lymphocytes as an immunoglobulin binding protein [34] and then later identified as tap42 in yeast [19]. Several studies have shown alpha4 to associate with all type 2A protein phosphatase C-subunits [11, 44, 54–56]. The association between alpha4 and the type 2A protein phosphatases was considered to be inhibitory in nature [35, 56]; however, this appears to be dependent on the substrate being dephosphorylated by the C-subunit/alpha4 heterodimer [63]. Recent studies have revealed that alpha4 plays a critical role in controlling the ubiquitination state and levels of PP2AC [41, 50, 82]. Hence, an additional consequence of the association between alpha4 and type 2A protein phosphatase catalytic subunits involves the “protection” of C-subunits from polyubiquitination and consequent 26S proteasome-mediated degradation [41]. A functional consequence of this type 2A catalytic subunit-alpha4 interaction was demonstrated by the genetic ablation of alpha4 protein expression, which induced the loss of type 2A C-subunit expression and apoptotic cell death in a number of cell types [41, 42].

Protein phosphatase 4 (PP4), the eukaryotic homologue of yeast Pph3p, is regulated by its association with a family of regulatory proteins termed R1–R4 [10, 26, 28, 40]. Evidence suggests that an active unit of the PP4 phosphatase can be comprised of either a simple heterodimer of PP4C and R1/R2 (low activity) or an active heterotrimer with any two of the aforementioned regulatory subunits [16, 26]. In addition, the R2 subunit is thought to confer cisplatin resistance in mammalian cells [29]. Furthermore, the R3 regulatory subunit can be subdivided into two closely related homologues α and β, of which the R3α isoform is overexpressed in breast and lung cancers [81]. The last PP4C regulatory subunit to have been characterised R4/KIAA1622 does not appear to “bridge” the association of PP4C with any of the other regulatory subunits [10].

The PP6C subunit was first cloned in budding yeast as sit4p [4]. Regulation of PP6C in mammalian cells is controlled by its association with SAP1-3 [47] and ANKRD28, 44 or 52 [77]. One of many several cellular functions ascribed to PP6C indicates that SAP1 facilitates PP6C-mediated dephosphorylation and activation of DNA protein kinase (DNA-PK) [31, 51], which then phosphorylates H2A.X at Ser139 [53, 60, 78]. Phosphorylated H2AX (γH2AX) is required for the recruitment of DNA repair proteins [61] and eventual repair of DNA double-strand breaks by non-homologous end-joining (NHEJ) [20]. SAP2 and SAP3 proteins have been suggested to play a role in cell cycle-related mTORC1-PP6 signalling events in yeast [52] and homology-directed repair of DNA in cancerous tissue [84]. The ANKRD proteins share >58% overall sequence identity, where the N-terminal 400 amino acids are almost 100% conserved and the C-terminal tail contains stretches of conserved/non-conserved amino acid sequences that contribute to the highest variability between isoforms [76]. Reported functionality of the ANKRD28 protein is limited to being involved in dephosphorylation of the inhibitory subunit of nuclear factor κB epsilon (IκΒε) in response to TNFα [76], and more recently, it was found to associate with the tumour suppressor protein BRCA1 [80]. The cellular functions for the other ANKRD regulatory proteins (44 and 52) remain poorly defined.

As in many other tissues, the regulation of PP2A in cardiac health and disease has been explored [18, 25, 57, 58], but relatively little is understood regarding the regulation of PP4C and PP6C in the heart. PP4C protein expression was first identified in cardiac tissue by Kloeker and colleagues albeit at very low levels in comparison to PP2AC [39]. However, abundant PP6C expression was identified in heart tissue almost 20 years ago by Bastians and Ponstingl [5]. Since these studies, very little has been reported regarding the expression/regulation of PP4C and PP6C in the healthy or diseased heart. The main objectives of the present study were to determine the expression and regulation of the type 2A protein phosphatase catalytic subunit/alpha4 intracellular signalling axis in the healthy and diseased myocardium.

## Methods and materials

Animal tissue used in this study was obtained in accordance with the UK Home Office Guidance on the Operation of the Animals (Scientific Procedures) Act 1986 (UK), the Directive of the European Parliament (2010/63/EU), and received approval by the local ethics review board at King’s College London. Healthy animals were killed by a schedule one procedure completed by a home office licensed individual, such that animal suffering was categorised as minimal.

### Materials

Sheep polyclonal anti-PP2AC, -PP4C, and -PP6C antibodies were described previously [39]. Primary antibodies to detect SAP1 (V-14), SAP2 (H-43), procaspase 3 (H-277), and actin (I-19) were purchased from Santa Cruz Biotechnologies (USA), whereas the anti-SAP3 antibody was purchased from Proteintech™ (USA). Antibody to detect ANKRD28 was purchased from Bethyl Laboratories (USA), whereas antibodies to detect ANKRD44 and ANKRD52 were purchased from LifeSpan Biosciences Inc (USA). Rabbit polyclonal anti-alpha4 antibody (A300-417A) was purchased from Bethyl Laboratories (USA), whereas the mouse monoclonal anti-alpha4 (5F6), rabbit monoclonal anti-γH2A.X (Ser139), rabbit polyclonal anti-H2A.X, and rabbit polyclonal anti-PARP1 antibodies were obtained from Cell Signaling Technology (USA). Whole rabbit IgG was purchased from Sigma-Aldrich (UK). HRP-conjugated donkey anti-goat and anti-sheep secondary antibodies were purchased from Santa Cruz Biotechnologies. HRP-conjugated donkey/goat anti-rabbit and sheep/horse anti-mouse secondary antibodies were purchased from either GE Healthcare (UK) or Cell Signaling Technology, respectively. Antibodies to phosphorylated (Ser63 and Ser68) or non-phosphorylated phospholemman were either custom-made and a kind gift from Professor MJ Shattock [24] or obtained from Abcam (#200204), respectively. Anti-actin primary antibody was detected using a donkey IRDye^®^680RD anti-goat secondary antibody purchased from LI-COR (UK). Protein A paramagnetic beads were purchased from Cell Signaling Technology. RNA extraction was performed using an RNeasy^®^Protect cell mini kit and QIAshredder homogenizer (Qiagen, UK). Agilent RNA 6000 Nano kit was purchased from Agilent Technologies, USA. Verified quantitative PCR primer pairs were obtained from Primerdesign Ltd., UK, and included: rat PP2ACα forward primer 5′-TTACCGAGAGCGTATCACCATA-3′ and reverse primer 5′-TTTCCGTATTTCCTTAAACACTCATC-3′; PP2ACβ forward 5′-TCGTGACTGGTTAAGGGAAAGG-3′ and reverse 5′-AAACTCCAACT CTATAATCCATGCC-3′; PP4C forward 5′-TGACATCCACGGACAATTCTATG-3′ and reverse 5′-CAGCAGGAGGAGGAAGGTTT-3′; PP6C forward 5′-GGCT TGTTCTTCCTAAAATGGC-3′ and reverse 5′-TTCCAAGAGCAGATCACAAA CATA-3′; and β-actin (accession number: NM_031144). All other qRT-PCR reagents (NanoScript 2 Reverse Transcription kit, Precision™2X qPCR Mastermix) were also purchased from Primerdesign Ltd., UK. ON-TARGETplus SMARTpool rat-specific siRNA to alpha4 and PP6C, as well as ON-TARGETplus non-targeting control pool siRNA, were purchased from GE Healthcare UK (Dharmacon).

### H9c2 cardiomyocyte cell culture

H9c2 cardiomyocytes were obtained from ATCC^®^ (#CRL-1446™) and grown in DMEM (Life Technologies) supplemented with 10% FBS (Life Technologies), penicillin 100 IU/mL, and streptomycin 100 μg/mL. Cells were grown at 37 °C in humidified 5% CO_2_/95% air until 80% confluent and were then either passaged or processed for analysis of gene expression by quantitative RT-PCR. Furthermore, H9c2 cardiomyocytes were also treated with either MG132 or H_2_O_2_ for 24 h followed by lysis with Laemmli sample buffer and western analysis. H9c2 cardiomyocytes cultured for PP6C and alpha4 knockdown studies were initially seeded at ~30% confluency and incubated with an appropriate amount of siRNA.

### Isolation and cell culture of neonatal rat ventricular myocytes (NRVMs)

Neonatal rat ventricular myocytes were isolated and cultured as previously described [64]. In brief, NRVM were isolated from 1–2-day-old Sprague–Dawley rats by collagenase-pancreatin digestion. Cells were plated at a density of ~2 × 10^6^ cells per well on gelatin-coated 6-well culture plates (Nunclon) and maintained in medium containing DMEM:M199 (4:1) supplemented with 5% FCS, 10% horse serum, and 1% penicillin/streptomycin for 24 h. Fibroblast growth was inhibited by the addition of 20 μM cytosine arabinoside (Sigma-Aldrich). NRVM were either cultured for 48 h prior to western analysis or processed for RNA extraction and analysis of gene expression by quantitative RT-PCR.

### Isolation and cell culture of adult rat ventricular myocytes (ARVMs)

ARVM were isolated from the hearts of adult male Wistar rats (200–250 g, B&K Universal Ltd.) by collagenase-based enzymatic digestion, as previously described [74]. ARVM were then allowed to rest for 2 h post-isolation to form a pellet, followed by a brief centrifugation at 50*g*. Storage medium was then removed and the ARVM pellet was resuspended in sterile PBS prior to being cultured according to previously described protocols [73, 74]. Freshly isolated ARVM were either cultured for 24 h prior to treatment and western analysis or processed for RNA extraction and analysis of gene expression by quantitative RT-PCR.

### Determination of type 2A protein phosphatase catalytic subunit gene expression by quantitative RT-PCR

Freshly isolated ARVM, cultured NRVM, or H9c2 cardiomyocytes were harvested and centrifuged at 100*g* for 3 min at room temperature. Cell pellets (1–2 × 10^6^ cells) were either immediately resuspended in RNAprotect cell reagent at room temperature following the manufacturer’s instructions and processed immediately for RNA extraction or stored long term at −20 °C. Total RNA was isolated from cells using the RNeasy protect cell mini kit according to the manufacturer’s protocol. Cellular homogenization was performed by QIAShredder spin columns. All RNA samples were subjected to only one freeze–thaw cycle. Total RNA purity and quantity was assessed by obtaining the RNA integrity number (RIN) from cardiomyocyte samples using the Agilent RNA 6000 Nano kit and the Agilent 2100 Bioanalyzer system (Agilent Technologies, USA). H9c2 and ARVM samples with a RIN between 9 and 10 and between 7 and 9, respectively, were selected for subsequent qPCR analysis [23]. Depending on the RNA concentration, 500 ng or 1 μg of total RNA was reverse transcribed into cDNA using oligo-dT primers via a two-step reverse transcription process using the NanoScript 2 reverse transcription kit (PrimerDesign Ltd., UK) and used according to the manufacturer’s instructions. cDNA was diluted to 5 ng/μL and stored at −20 °C. All qPCR reactions were performed in a Stratagene Mx3005P qPCR system (Agilent Technologies, USA) using Precision™2X MasterMix according to the manufacturer’s instructions. Each reaction contained 5 μL diluted cDNA (25 ng in total), 10 μL Precision™2× qPCR MasterMix, 1 μL primer mix (300 nM final concentration), and 4 μL RNAse/DNAse free water. A non-reverse transcriptase control (NRT) and a non-template control (NTC) were included as negative controls for each gene. The qPCR conditions were as follows: 10 min at 95 °C, followed by 40 cycles of 15 s at 95 °C and 1 min at 60 °C. Fold change in mRNA levels was calculated using the comparative *C*_T_ method [70] and expressed relative to PP2ACα mRNA levels. Values were expressed as the averaged quantification cycle (*C*_q_) value of each gene normalised to the averaged *C*_q_ values for reference gene β-actin. The mean *C*_q_ accepted standard deviation (SD) was ≤0.15 cycles. All data are presented in accordance with MIQE guidelines on the minimum information for publication of quantitative real-time PCR experiments [9].

### Knockdown of protein expression in H9c2 cardiomyocytes by siRNA

H9c2 cardiomyocytes were cultured as described above in 6-well plates with a subcultivation ratio of 1:10 and were then maintained in DMEM culture medium supplemented with 10% FBS, penicillin 100 IU/mL and streptomycin 100 μg/mL at 5% CO_2_/37 °C until they reached 30% confluency. Cultured H9c2 cardiomyocytes were incubated with ON-TARGETplus SMARTpool rat alpha4, PP6C specific siRNA, or ON-TARGETplus non-targeting control pool siRNA. The transfection mix was prepared using a ratio of 1 μL of DharmaFECT(#1):50 pmol of siRNA and allowed to incubate on ice for 20 min to form siRNA:DharmaFECT complexes. The transfection mix was then combined with an appropriate volume of antibiotic-free DMEM containing 10% FBS to achieve a final siRNA concentration of 50 nM. Transfected H9c2 cardiomyocytes were incubated at 37 °C in 5% CO_2_ for 1–8 days. The transfection medium was replaced and cells re-transfected every 4 days when silencing PP6C protein expression for up to 8 days. The cells were then lysed with 1× Laemmli sample buffer for subsequent western analysis.

### Pressure overload-induced murine hypertrophy

Myocardial hypertrophy was induced as previously described by Boguslavskyi et al. [6]. In brief, pressure overload was induced by suprarenal transaortic constriction (TAC) in 6-week-old C57BL/6J mice (20–22 g). The reproducibility of the transaortic constriction was assured by placing a suture around the abdominal aorta and a 28-gauge blunted needle, which was then subsequently removed. The suture remained in place for 4 weeks after which the animals were killed and the hearts excised, whereupon the LV free wall was dissected away and processed for subsequent protein analysis. Hypertrophic growth was expressed as a ratio of LV tissue to tibia length. Sham animals were treated identically except that the suture around the aorta was not tied in place. For all surgical procedures, mice were anaesthetized by inhalation of isoflurane/O_2_ mixture (2/98%). Adequacy of anaesthesia was controlled by monitoring corneal reflex and respiration (rate, depth, and pattern of breathing). Acute postoperative analgesia was a single buprenorphine (Vetergesic 0.3 mg/mL solution) injected i.p. at dose 20 mg/kg. Euthanasia was performed by overdose of isoflurane. Death was confirmed by monitoring cardiac activity and respiration. Heart excision for homogenisation and tissue lysate preparation was performed after terminal anaesthesia by i.p. injection of pentobarbital (Pentoject 200 mg/mL solution) at dose 300 mg/kg and heparin (150 U).

### Preparation of murine ventricular tissue for protein analysis

The excised heart was rapidly flushed with ice-cold PBS to remove any residual blood and extraneous tissue was then removed. The LV free wall was dissected away and immediately placed into liquid nitrogen for short-term storage. LV tissue was homogenised on ice (100 mg tissue/ml of buffer) in ice-cold homogenisation buffer containing; 20 mM MOPS, 140 mM NaCl, 5 mM KCl, 1 mM EDTA, phosphatase inhibitor cocktail #3 (Sigma-Aldrich, UK), and protease inhibitor cocktail set 3 (Calbiochem, UK). Homogenates were diluted (1:1) with 2× Laemmli sample buffer for subsequent western analysis or frozen at −80 °C for subsequent immunoprecipitation of alpha4 protein. The protein content of tissue homogenates was adjusted to 500 μg/mL with ice-cold lysis buffer; 150 mM NaCl, 1 mM EDTA, 1 mM EGTA, 2.5 mM sodium pyrophosphate, 1 mM β-glycerophosphate, 1 mM Na_3_VO_4_, 20 mM Tris–HCl, 1% Triton X-100, 1 μg/mL leupeptin, mini-Complete protease inhibitor cocktail (Roche, UK), and pH 7.5. Cell lysates were incubated on ice for 5 min and centrifuged at 14,000*g* at 4 °C for 30 min to separate the triton-soluble fraction (supernatant) from the triton-insoluble particulate fraction (pellet). An aliquot of the solubilised fraction was removed for subsequent western analysis of protein expression in the input pre immunoprecipitation (pre-IP) lysate. To the remaining solubilised lysate 2 μL (1 μg/μL) of rabbit IgG, 2 μL PBS, or 2 μL (1 μg/μL) of rabbit polyclonal anti-alpha4 antibody was added. The addition of rabbit IgG or PBS was to test for non-specific binding of IgG or protein A to alpha4 protein, respectively. All samples were then incubated with gentle inversion overnight at 4 °C. Each tube was then incubated with 40 μL of protein A paramagnetic bead slurry for 2 h at 4 °C. The immunoprecipitate complex was then washed three times with ice-cold cell lysis buffer and then resuspended in 50 μL of 3× Laemmli sample buffer.

### Western analysis

Cultured cells were rinsed with ice-cold PBS and then lysed with 1× Laemmli sample buffer unless otherwise stated. Western analysis was carried out as previously described [75]. In brief, protein samples were separated by 10.5–15% SDS-PAGE, transferred to PVDF (0.2 or 0.45 μm pore size) or nitrocellulose membranes where appropriate and probed with primary antibodies. To confirm equal protein loading in each sample, actin content was determined using an anti-actin primary antibody which was detected using a donkey anti-goat IRDye^®^680RD secondary antibody followed by image acquisition and quantification using an LI-COR Odyssey CLx system. Where appropriate, primary antibodies were detected using donkey anti-sheep or anti-goat; donkey or goat anti-rabbit; sheep or horse anti-mouse secondary antibodies (1:1000) linked to HRP. Specific protein bands were detected by enhanced chemiluminescence (GE Healthcare, UK) and band intensity was quantified using a calibrated GS-800 densitometer and Quantity One^®^ 1-D analysis software v4.6.2 (Bio-Rad, UK). Equal protein loading was determined either by quantifying the content of non-phosphorylated protein (where appropriate) or actin within each sample, which unless otherwise stated was used to normalise the expression of all other proteins of interest.

### Statistical analysis

Data are presented as mean ± SEM. Where appropriate, data were subjected to either an unpaired Student’s *t* test or ANOVA (GraphPad Prism v6.0.1) to test for significant differences (*P* < 0.05) between groups. Further multiple group comparison was completed using either a Dunnett’s or Tukey’s modified *t* test.

## Results

### Expression of type 2A phosphatase catalytic subunits in cardiomyocytes

**Fig. 1:**
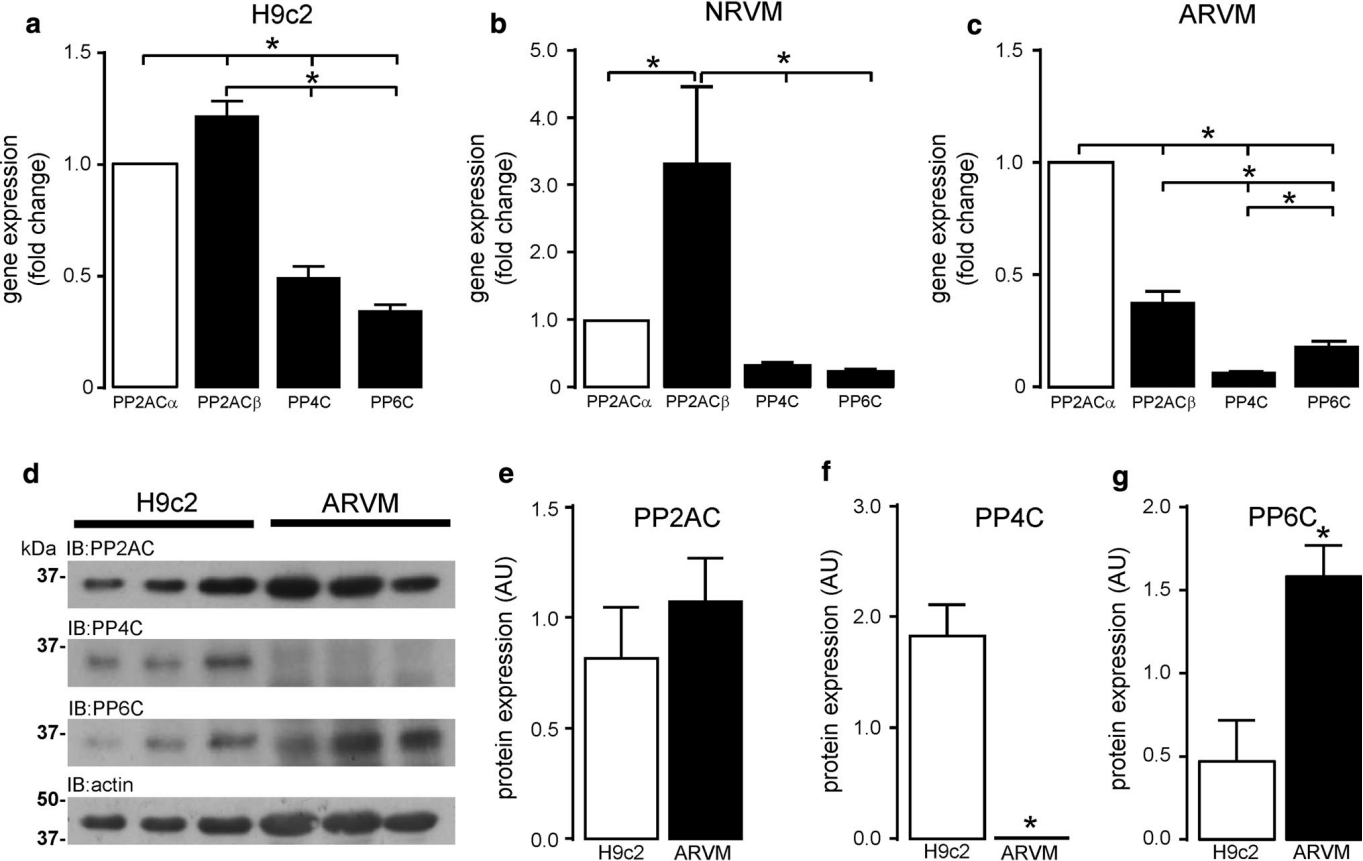
Expression of type 2A protein phosphatase catalytic subunits in H9c2 cardiomyocytes and ARVM. qRT-PCR determination of type 2A protein phosphatase catalytic subunit mRNA expressed as fold change relative to PP2ACα and normalised to β-actin in cultured H9c2 cardiomyocytes (**a**) and freshly isolated ARVM (**b**). Protein expression of type 2A protein phosphatase catalytic subunits was determined by western analysis with subunit-specific antibodies to PP2AC, PP4C, and PP6C (**c**). Levels of phosphatase catalytic subunit expression from cultured H9c2 and freshly isolated ARVM lysates were quantified by densitometry and normalised to actin (**d**–**f**). All data represent mean values ± SEM of three individual experiments, *P < 0.05

The expression of type 2A protein phosphatase catalytic subunits was determined at both the mRNA and protein level in rat embryonic H9c2 cardiomyocytes, neonatal rat ventricular myocytes (NRVM), and adult rat ventricular myocytes (ARVM). Data confirm that gene transcription of all type 2A protein phosphatases was active in H9c2 cardiomyocytes (Fig. 1a), NRVM (Fig. 1b), and ARVM (Fig. 1c), as mRNA was detected for all type 2A phosphatases. PP2ACβ mRNA expression seemed to be most abundant in both H9c2 cardiomyocytes and NRVM, whereas PP2ACα mRNA appeared to be most abundant in ARVM. PP4C mRNA expression was >14-fold less than PP2ACα mRNA in ARVM. All three type 2A catalytic subunits were expressed at the protein level in H9c2 cardiomyocytes (Fig. 1c–f) and NRVM (Supplemental Figure S1); however, we could not detect any PP4C protein expression in ARVM (Fig. 1f). Furthermore, PP6C expression was significantly higher in ARVM than in H9c2 cardiomyocytes (Fig. 1g). The selectivity of the antibodies towards specific type 2A protein phosphatase catalytic subunits was confirmed by siRNA-mediated knockdown of PP2ACα (Supplemental Figure S2), PP2ACβ (Supplemental Figure S3), PP4C (Supplemental Figure S4), or PP6C (Supplemental Figure S5) in H9c2 cardiomyocytes followed by western analysis to determine the expression levels of the other type 2A protein phosphatase catalytic subunits. These data suggest that there is differential expression of PP2AC, PP4C, and PP6C at both the mRNA and protein level in cardiac tissue.

### Role of the 26S proteasome in type 2A protein phosphatase catalytic subunit expression in ARVM

**Fig. 2:**
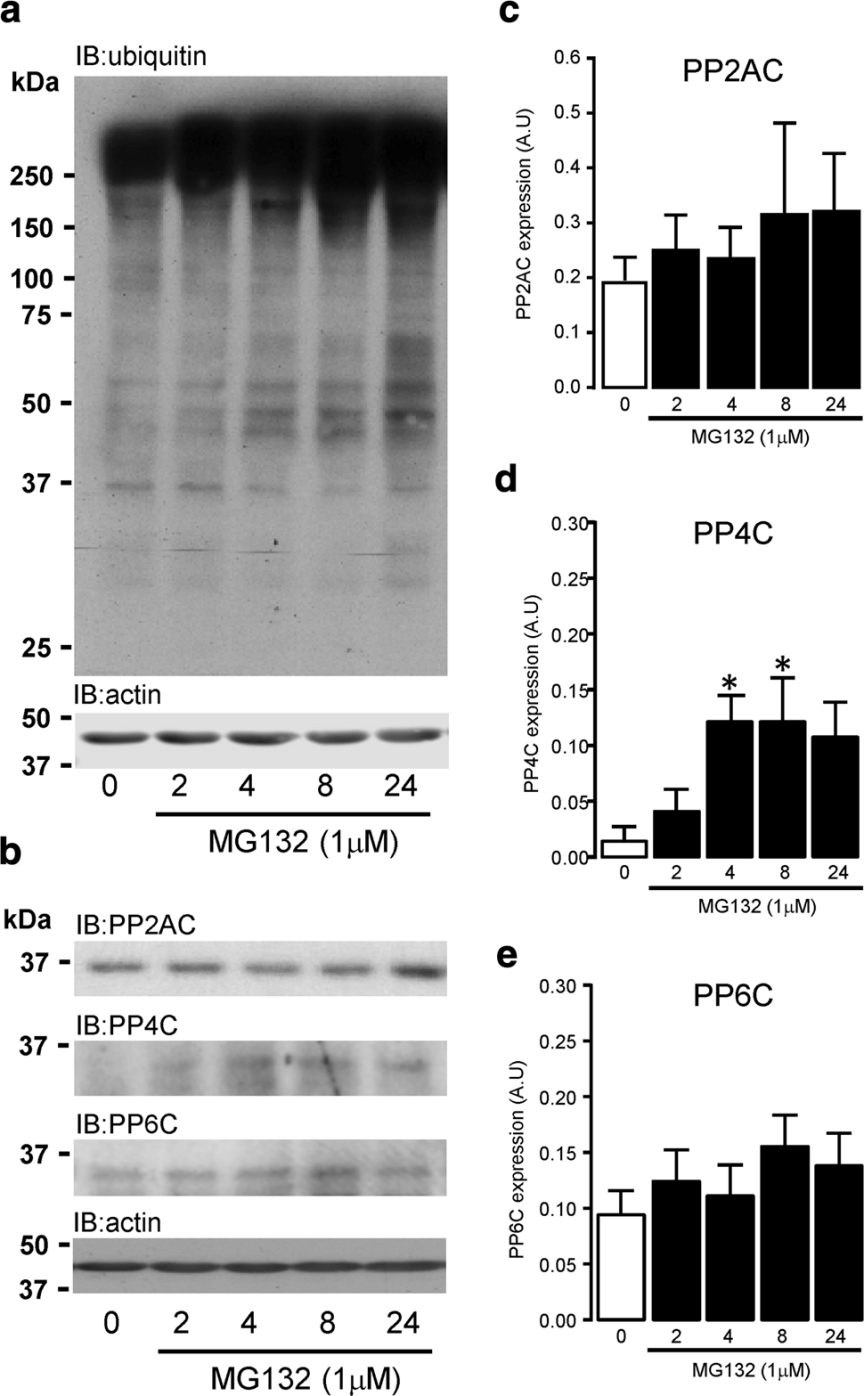
Proteasomal degradation of type 2A protein phosphatase catalytic subunits in ARVM. Cultured ARVM were exposed to MG132 (1 μM) for 0, 2, 4, 8, and 24 h. Ubiquitination of proteins was determined by western analysis using an anti-ubiquitin antibody (**a**). Effect of 26S proteasome inhibition on the expression of type 2A protein phosphatase catalytic subunits was determined by western analysis using subunit-specific antibodies to PP2AC, PP4C, and PP6C (**b**) and then quantified by densitometry (**c**–**e**). Data are representative of three individual experiments and represent mean values ± SEM, *P < 0.05 vs. untreated control

One possible explanation why PP4C protein expression was undetectable in cultured ARVM, despite expression of PP4C mRNA, could be that it is subject to excessive proteasomal degradation. To test this idea, we treated ARVM with MG132 (1 μM) to inhibit proteasome activity, which was confirmed by progressively elevated levels of protein ubiquitination from 0 to 24 h (Fig. 2a). The western analysis data (Fig. 2b) and quantification by densitometry (Fig. 2c–e) suggest that the expression level of type 2A protein phosphatases in ARVM was differentially regulated by the 26S proteasome. The expression of PP2AC and PP6C protein was not significantly altered by MG132-mediated inhibition of the 26S proteasome for 24 h. However, PP4C expression which was undetectable in untreated ARVM, increased significantly (*P* < 0.05) following 4 and 8 h of 26S proteasome inhibition with 1 μM MG132 (Fig. 2d). These data indicate that the apparent absence of PP4C protein expression in the adult myocardium may have been due (in part) to proteasome-mediated degradation of PP4C.

### Regulation of type 2A phosphatase catalytic subunit expression by alpha4

**Fig. 3:**
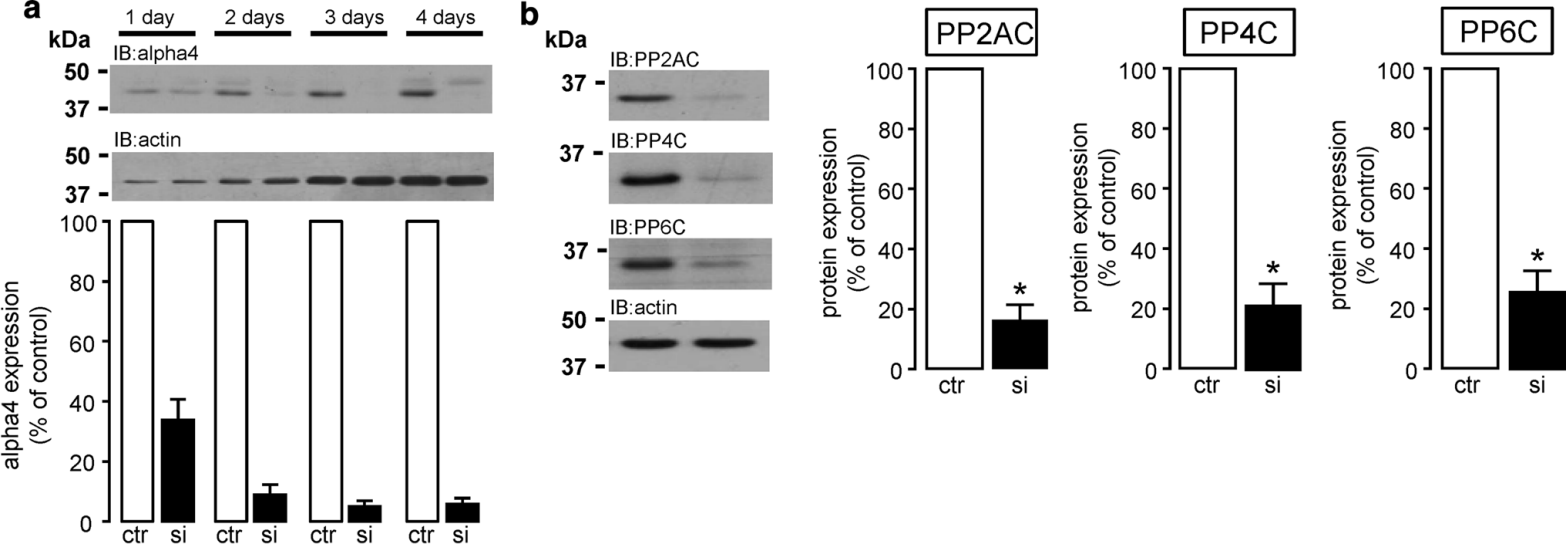
Regulation of type 2A protein phosphatase catalytic subunit expression by alpha4 in cardiomyocytes. H9c2 cardiomyocytes were incubated with either 50 nM non-targeting control siRNA (ctr) or 50 nM rat alpha4-specific siRNA (si) for 1–4 days to silence the expression of alpha4 protein which was confirmed by western analysis with a polyclonal alpha4 specific antibody (**a**). Expression of type 2A protein phosphatase catalytic subunits (PP2AC, PP4C and PP6C) was then determined in H9c2 cardiomyocytes using type 2A phosphatase catalytic subunit-specific antibodies and western analysis following siRNA-driven knockdown of alpha4 protein expression for 4 days (**b**). Equal protein loading was confirmed by the determination and then normalisation to actin in each sample. All data are representative of four individual experiments and represent mean values ± SEM, *P < 0.05 vs. ctr

To investigate whether the non-catalytic type 2A protein phosphatase regulatory protein alpha4 modulates type 2A protein expression in cardiomyocytes as previously reported in mouse embryonic fibroblasts [41], the expression of alpha4 protein was knocked down in H9c2 cardiomyocytes using rat alpha4-specific siRNA (si) for 1–4 days. Alpha4 protein expression was significantly (*P* < 0.05) decreased by ~95% following 4 days of transfection with alpha4-specific siRNA when compared to control cells (Fig. 3a). Interestingly, knockdown of alpha4 in H9c2 cardiomyocytes significantly (*P* < 0.05) reduced the expression of all type 2A protein phosphatase catalytic subunits (PP2AC, PP4C, and PP6C) by 84, 78, and 74%, respectively, when compared to non-targeting control (Fig. 3b). These data confirm that alpha4 is, indeed, involved in the regulation of type 2A protein phosphatase expression/biogenesis in cardiomyocytes.

### Expression of the type 2A phosphatase-alpha4 signalling axis in left ventricular hypertrophy

**Fig. 4:**
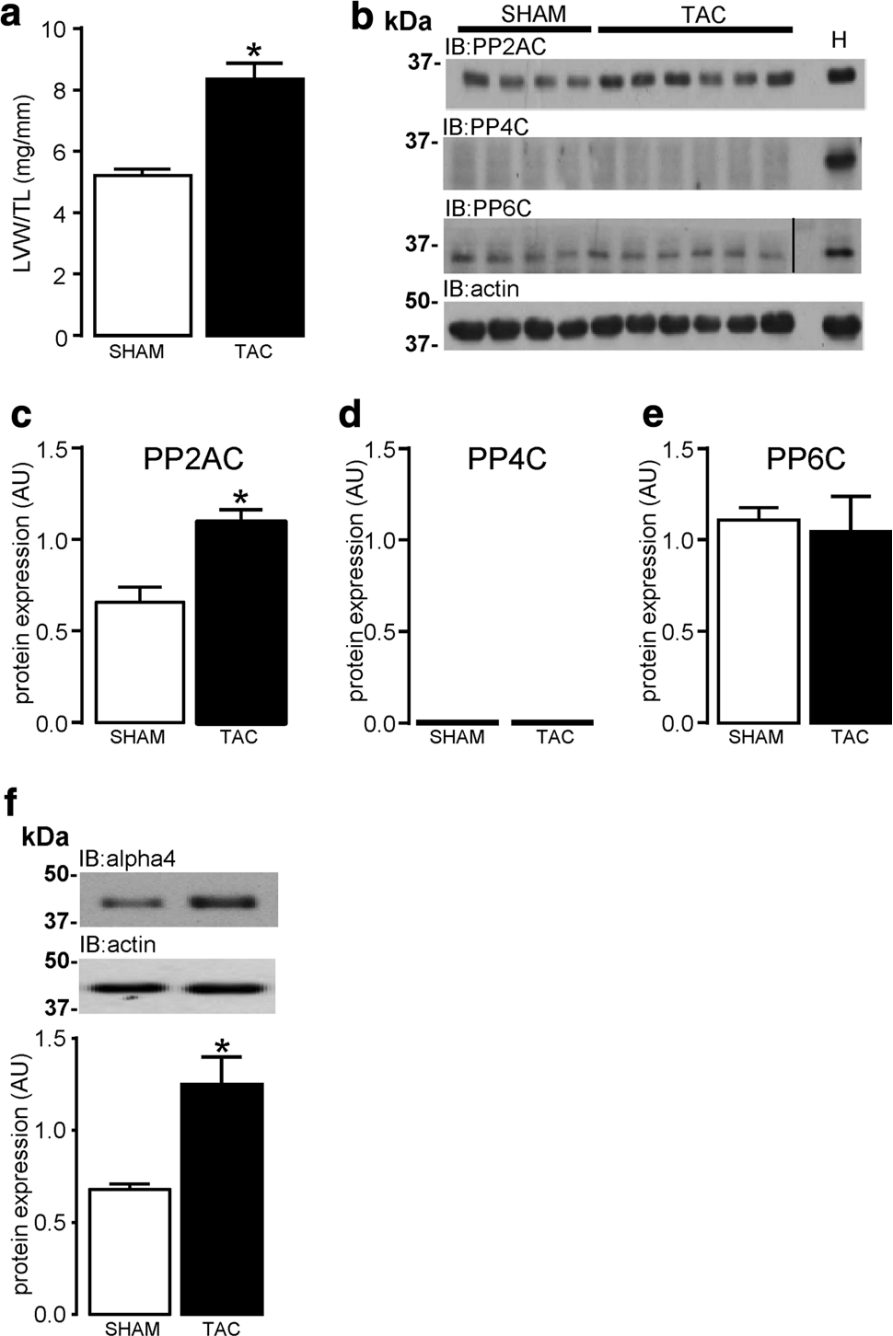
Expression of type 2A protein phosphatase catalytic subunits and alpha4 in LV hypertrophied tissue. Murine LV hypertrophy expressed as LV weight (LVW)/tibia length (TL) was induced by 28 days of TAC-mediated pressure overload (**a**). Protein expression and quantification of type 2A protein phosphatase catalytic subunits (PP2AC, PP4C, and PP6C) in LV tissue from both SHAM (*n* = 4) and TAC (*n* = 6) operated mice, was determined by western analysis using subunit-specific antibodies, where “H” indicates an H9c2 lysate positive control sample. PP6C immunoblot contains a splice (*black line*) to maintain formatting within the set of data (**b**–**e**). Protein expression of alpha4 in SHAM (*n* = 4) and TAC (*n* = 6) operated LV tissue was determined by western analysis with a polyclonal antibody to alpha4 (**f**). Protein expression was normalised to actin in each sample. All data represent mean values ± SEM, *P < 0.05 vs. SHAM

Four weeks of TAC-mediated pressure overload induced a ~60% increase in LV weight when expressed relative to tibia length (Fig. 4a). Further indices of hypertrophy (heart weight/body weight, heart weight/tibia length, and LV weight/body weight) were also determined (Supplemental Figure S6). Western analysis revealed that the expression of type 2A protein phosphatases was differentially altered in LV hypertrophied tissue (Fig. 4b). PP2AC protein expression in LV hypertrophied tissue was significantly (*P* < 0.05) higher (1.7-fold) than in non-hypertrophied LV SHAM control tissue (Fig. 4c). However, expression of PP4C was undetectable in mouse LV tissue (Fig. 4d), again confirming the absence of PP4C protein expression in adult myocardial tissue as previously observed in ARVM (Fig. 1e). No differences in PP6C protein expression were observed in lysates of LV tissue obtained from hearts of SHAM and TAC-operated mice (Fig. 4e). Interestingly, expression of alpha4 protein was significantly (*P* < 0.05) elevated (1.8-fold) in hypertrophied LV tissue when compared to LV tissue obtained from SHAM control hearts (Fig. 4f).

### Expression of PP6C regulatory proteins in left ventricular hypertrophy

**Fig. 5:**
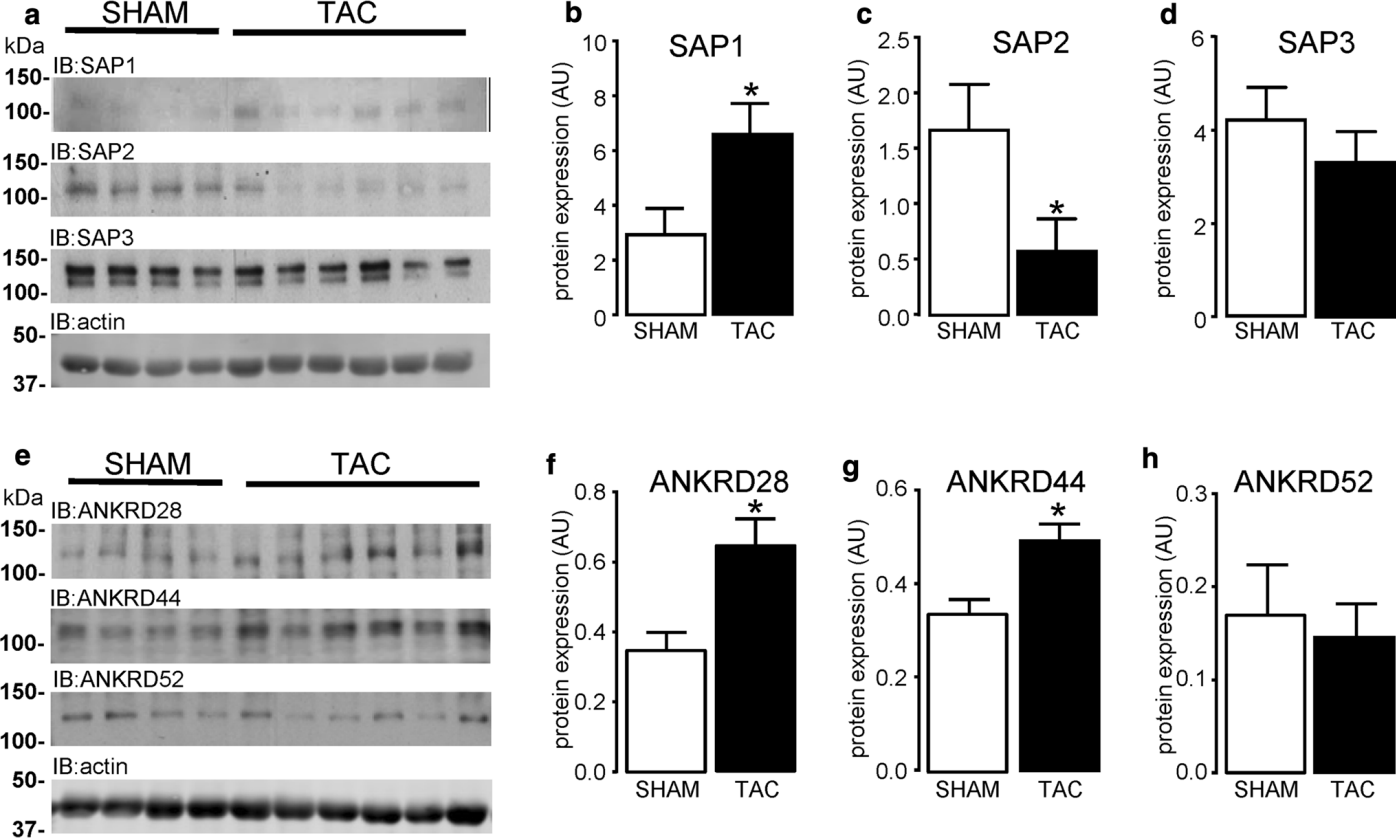
Expression of PP6C regulatory proteins in LV hypertrophied tissue. The expression of PP6C regulatory subunits SAP1-3 and ANKRD28/44/52 proteins was determined in LV tissue obtained from both SHAM (*n* = 4) and TAC (*n* = 6) operated mice, by western analysis with antibodies specific to SAP1-3 (**a**–**d**) and ANKRD28/44/52 (**e**–**h**) proteins. Protein expression was normalised to actin in each sample. All data represent mean values ± SEM, *P < 0.05 vs. SHAM

The regulation of PP6C subcellular localisation and activity is achieved by the association of the C-subunit with an SAP and ANKRD protein [77]. Data in Fig. 5a show changes in SAP1-3 protein expression, in response to TAC-induced LV hypertrophy. Quantification of these data revealed that SAP1 (Fig. 5b) and SAP2 (Fig. 5c) protein expression was either significantly (*P* < 0.05) increased (2.2-fold) or decreased (2.8-fold) in the hypertrophied left ventricle, respectively. SAP3 protein expression, unlike SAP1 and SAP2, was unchanged (Fig. 5d). Furthermore, expression of the PP6C regulatory proteins ANKRD28/44/52 in LV hypertrophied tissue was also differentially altered (Fig. 5e). Expression of ANKRD28 (Fig. 5f) and ANKRD44 (Fig. 5g) proteins was both significantly (*P* < 0.05) elevated 1.9- and 1.5-fold, respectively, whereas expression of ANKRD52 protein (Fig. 5h) remained unchanged in hypertrophied LV tissue. Despite the protein expression of PP6C not being altered in hypertrophied LV tissue (Fig. 4e), these data support a novel observation which suggests that there are differential changes in the expression of the PP6C regulatory SAP and ANKRD proteins in hypertrophied LV tissue.

### Association of alpha4 protein with type 2A protein phosphatase catalytic subunits in hypertrophied left ventricular tissue

**Fig. 6:**
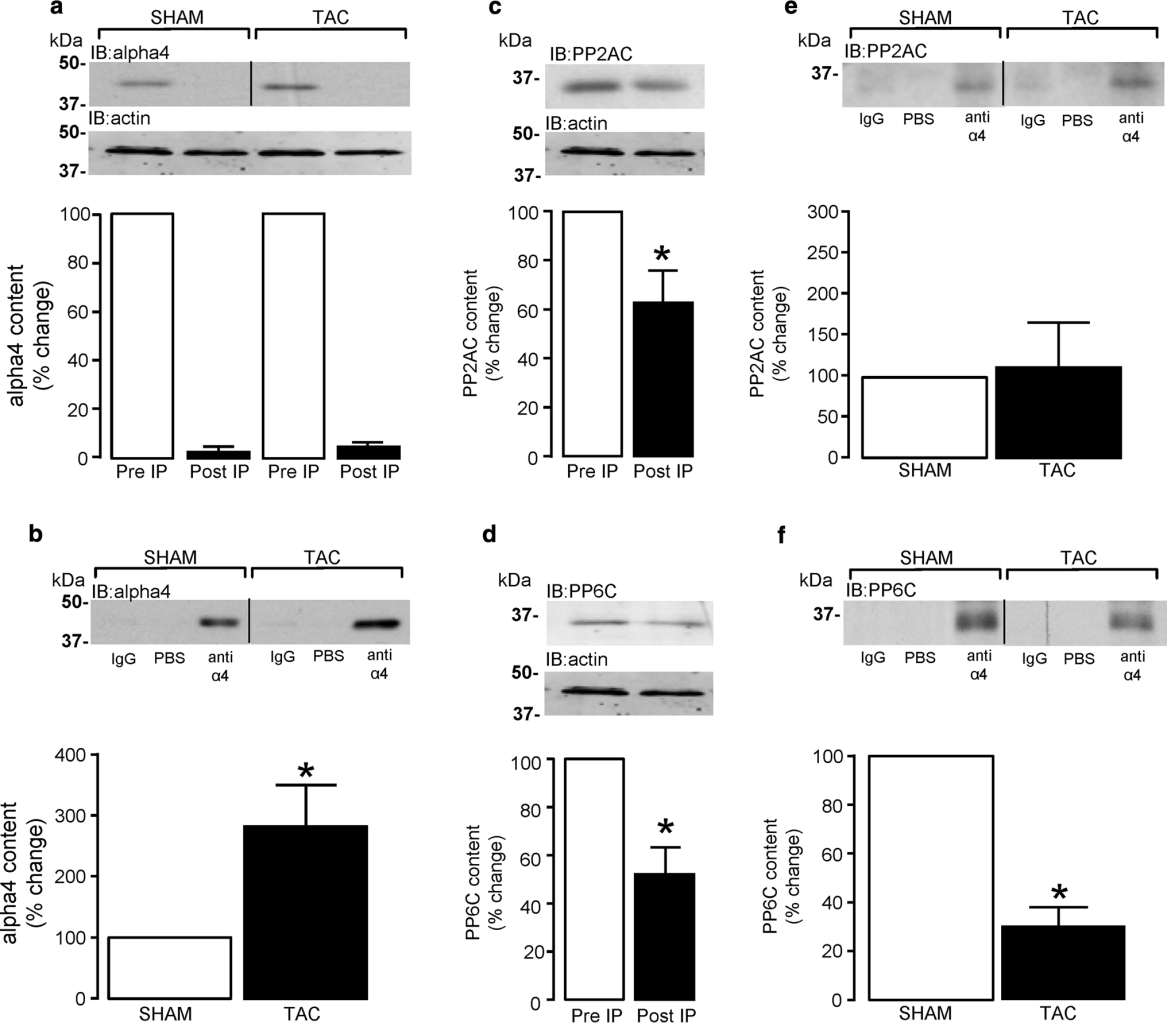
Association of PP2AC and PP6C with alpha4 in LV hypertrophied tissue. Immunoprecipitation of alpha4 protein and association with PP2AC or PP6C in LV tissue obtained from SHAM (*n* = 4) and TAC (*n* = 4) operated mice was performed using a polyclonal anti-alpha4 antibody in combination with protein A paramagnetic beads. Alpha4 protein content in the input pre-immunoprecipitate (pre-IP) and post-immunoprecipitate (post-IP) lysate was determined by western analysis using a monoclonal anti-alpha4 antibody (**a**). Alpha4 protein content in control (rabbit IgG and PBS) and anti-alpha4 immunoprecipitates from LV tissue lysate of SHAM (*n* = 4) and TAC (*n* = 4) operated mice was determined by western analysis using a monoclonal anti-alpha4 antibody and quantified by densitometry (**b**). PP2AC and PP6C protein content in lysates before (pre-IP) and after (post-IP) immunoprecipitation of alpha4 protein was determined by western analysis with subunit-specific anti-PP2AC (**c**) and anti-PP6C (**d**) antibodies. PP2AC and PP6C protein content in control (rabbit IgG and PBS) and anti-alpha4 immunoprecipitates from LV tissue lysate of SHAM (*n* = 4) and TAC (*n* = 4) operated mice was determined by probing immunocomplexes with subunit-specific anti-PP2AC (**e**) and anti-PP6C (**f**) antibodies. Immunoprecipitated PP2AC and PP6C were normalised to the alpha4 content of the immunoprecipitates and expressed relative to SHAM control tissue. PP2AC and PP6C immunoblots were spliced (*black line*) to aid clarity. *P < 0.05 vs. SHAM

The association of type 2A protein phosphatase catalytic subunits with alpha4 was investigated through co-immunoprecipitation of alpha4 and C-subunit protein complexes from SHAM- or TAC-operated mouse LV tissue lysates using a polyclonal alpha4 antibody. The protocol that we developed for immunoprecipitating alpha4 from LV tissue lysate depleted >95% of alpha4 protein from cell lysates (Fig. 6a). Furthermore, significantly (*P* < 0.05) higher alpha4 content was detected in immunocomplexes from LV hypertrophied tissue when compared to SHAM control LV tissue (Fig. 6b), probably due to the significantly higher level of alpha4 protein expression in LV tissue. Consequently, to accurately determine whether the association of PP2AC or PP6C protein with alpha4 protein was altered in hypertrophied tissue, the quantitation of PP2AC and PP6C in the alpha4 immunocomplex was normalised to the amount of alpha4 protein present. Immunoprecipitation of alpha4 protein from murine LV tissue significantly (*P* < 0.05) reduced the content of both PP2AC protein (Fig. 6c) and PP6C protein (Fig. 6d) in the post-IP lysate to 62.8 ± 13.2 and 52.0 ± 11.4%, respectively, when compared to the pre-IP “input” lysate. The alpha4 immunocomplexes were then probed with a PP2AC subunit-specific antibody and although the alpha4 immunocomplexes contain PP2AC protein, no differences in the association between PP2AC and alpha4 protein were observed between SHAM and TAC (hypertrophied) LV tissue (Fig. 6e). Hence, our data suggest that even though alpha4 protein associates with a significant proportion of PP2AC protein (37%) in cardiac tissue, this association is not affected by pressure overload-induced LV hypertrophy. Furthermore, when the same alpha4 immunoprecipitates from LV tissue lysates of SHAM- and TAC-operated mice were probed for PP6C content, we observed significantly (*P* < 0.05) less PP6C protein (30.4 ± 7.6%) within the immunoprecipitates from hypertrophied tissue, when compared to SHAM control tissue (Fig. 6f). These data not only show that alpha4 protein associates with a significant proportion (48%) of PP6C protein in cardiac tissue, but that this association is significantly reduced in hypertrophied tissue. Hence, our novel data demonstrate that alpha4 protein differentially associates with PP2AC and PP6C catalytic subunits in hypertrophied LV tissue.

### Regulation of phospholemman phosphorylation by type 2A protein phosphatases in cardiomyocytes

**Fig. 7:**
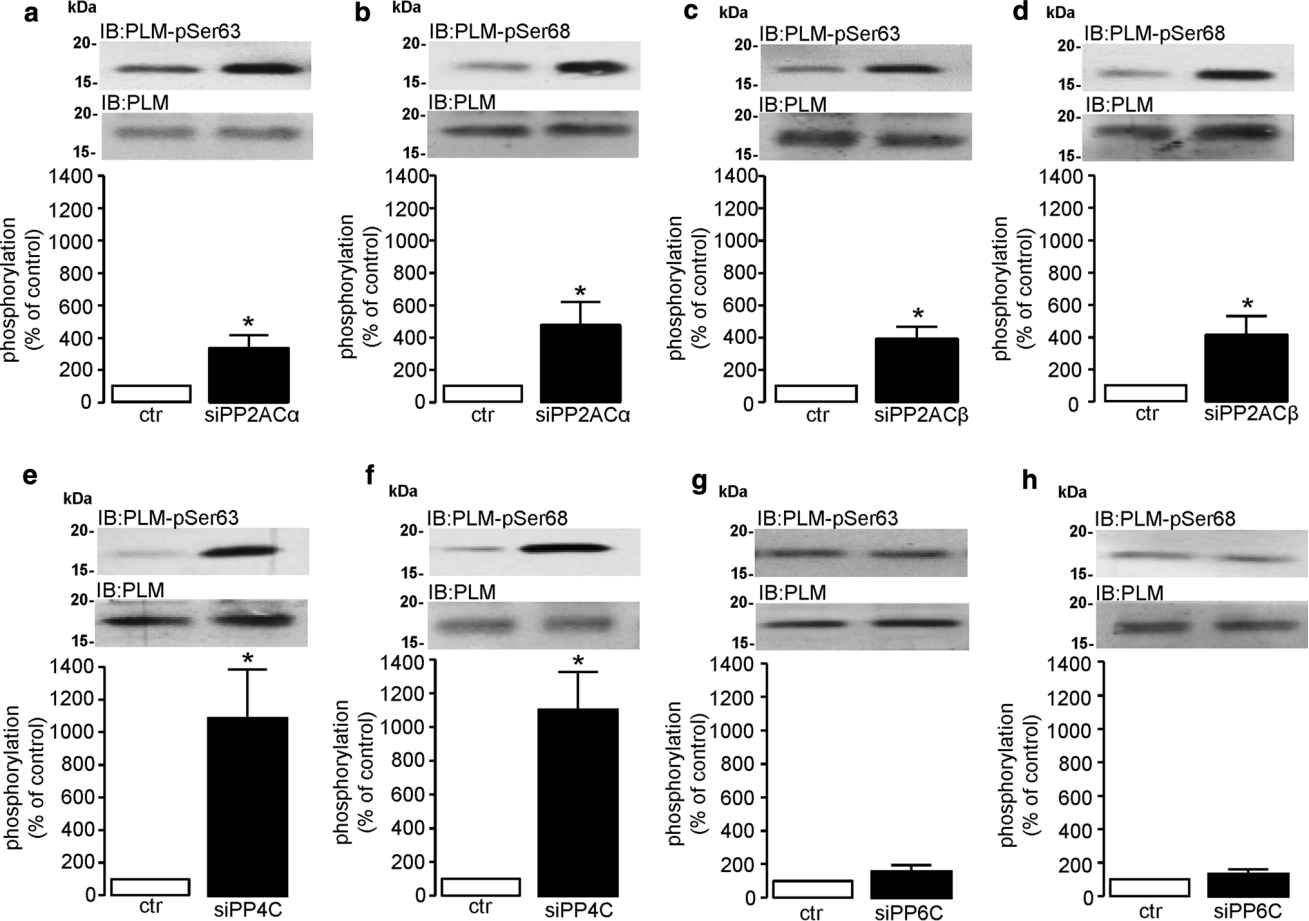
Regulation of phospholemman phosphorylation by type 2A protein phosphatases in cardiomyocytes. H9c2 cardiomyocytes were transfected with non-targeting control siRNA (ctr), rat-specific PP2ACα-siRNA (siPP2ACα), PP2ACβ-siRNA (siPP2ACβ), PP4C-siRNA (siPP4C), or PP6C-siRNA (siPP6C) for 4 days. Phosphorylation of PLM at Ser63 (**a**, **c**, **e**, **g**) or Ser68 (**b**, **d**, **f**, **h**) was determined by standard western analysis using rabbit polyclonal anti-phospho-PLM-Ser63, anti-phospho-PLM-Ser68, and rabbit monoclonal anti-PLM antibodies. Protein levels were quantified by densitometry and were normalised to total PLM in each sample. All data represent mean values ± SEM of six individual experiments. Statistical comparison was made by a two-tailed unpaired Student’s *t* test; *P < 0.05 vs. ctr

Phospholemman (PLM) is a phosphoprotein and a negative regulator of sarcolemmal Na^+^/K^+^-ATPase (sodium pump) activity in the heart (as reviewed by Pavlovic et al. [62]). Phosphorylation of PLM at Ser63/Ser68 underpins the inhibition of its activity towards the sodium pump and its hypophosphorylation at these two sites has been implicated to play a role in the development of cardiac hypertrophy [6]. Historically, the use of non-specific and non-selective protein phosphatase inhibitors has been used to implicate a role for PP2AC in dephosphorylating PLM in the heart [59]. However, using subunit selective siRNA molecules to knockdown the expression of each individual type 2A catalytic subunit, our study shows that PLM phosphorylation at both Ser63 and Ser68 was significantly elevated when expression of either alpha (331.7 ± 79.0, 471.0 ± 145.2%) or beta (386.4 ± 76.3, 408.4 ± 119.2%) PP2AC isoforms was knocked down (Fig. 7a–d), respectively. In addition, the knockdown of PP4C expression significantly (*P* < 0.05) elevated Ser63 (1082.0 ± 303.5%) and Ser68 (1101.0 ± 225.9%) phosphorylation. However, the phosphorylation of PLM at Ser63 (Fig. 7g) and Ser68 (Fig. 7h) was unaltered when PP6C protein expression was attenuated by siRNA-mediated knockdown. Therefore, using selective tools to abrogate the expression of individual type 2A catalytic subunits, our data suggest that not only do both isoforms of PP2AC regulate the phosphorylation status of PLM in cardiomyocytes, but so does PP4C. Despite PP6C protein being highly expressed in the myocardium, it does not appear to have a role for modulating the phosphorylation status of PLM in the myocardium.

### Regulation of H2A.X phosphorylation by H_2_O_2_ in cardiomyocytes

**Fig. 8:**
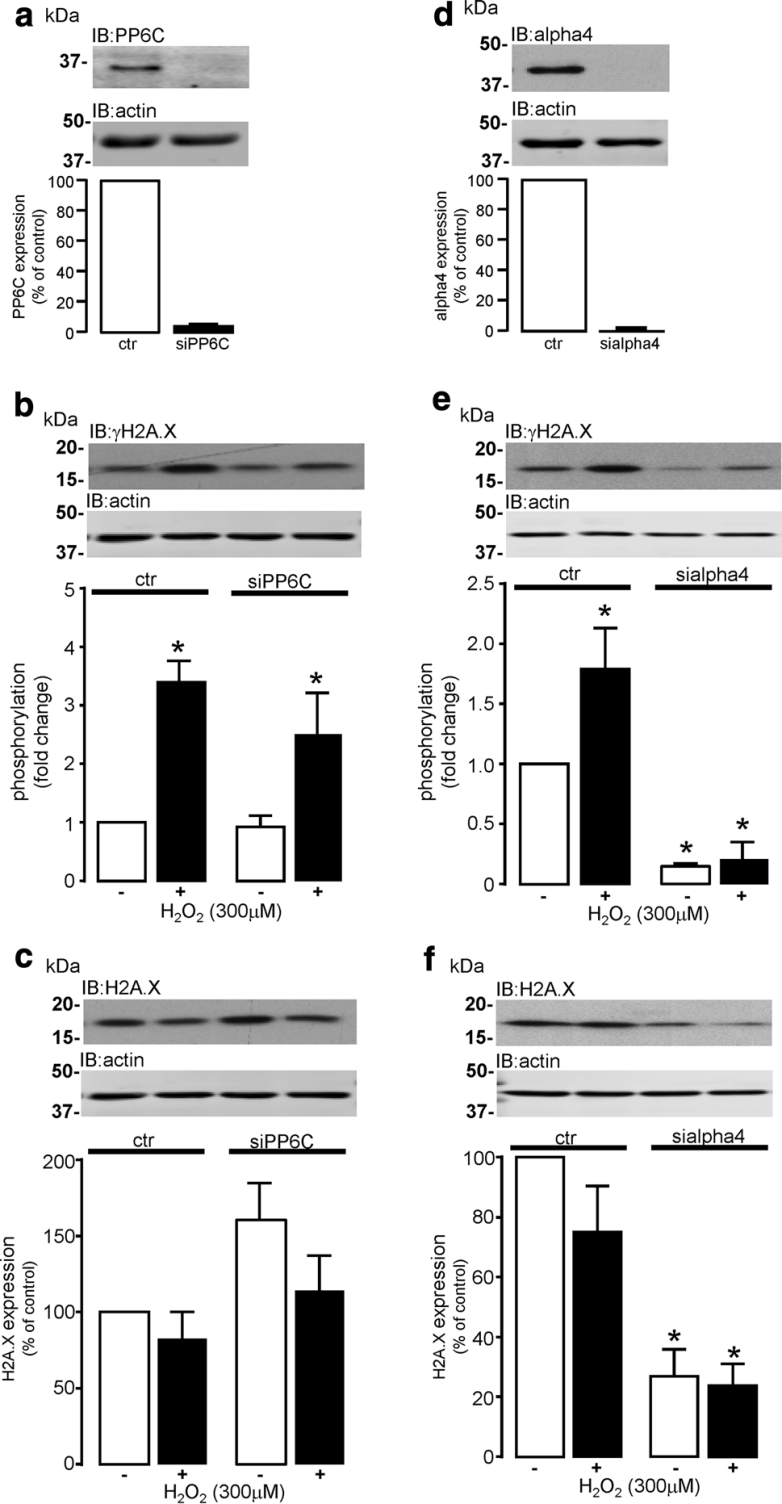
Regulation of H_2_O_2_-induced H2A.X phosphorylation by PP6C or alpha4 in cardiomyocytes. H9c2 cardiomyocytes were transfected with non-targeting control siRNA (ctr) or rat PP6C-specific siRNA (si) to knockdown the expression of PP6C protein, which was confirmed by standard western analysis using an antibody specific to PP6C (**a**). Cardiomyocytes transfected with non-targeting control siRNA or PP6C siRNA were then exposed to vehicle control (−) or H_2_O_2_ (+) for 24 h and phosphorylation of H2A.X at Ser139 (γH2A.X) was determined by standard western analysis (**b**). Total H2A.X protein expression was also determined by standard western analysis in cardiomyocytes following knockdown of PP6C (**c**). Cardiomyocytes were also transfected with either non-targeting control siRNA (ctr) or rat alpha4-specific siRNA (si) and treated as described above (**d**–**f**). Protein expression was quantified by densitometry and where appropriate was then normalised to actin in each sample. All data represent mean values ± SEM of four individual experiments, *P < 0.05 vs. untreated ctr

Oxidative stress is considered to be an associative risk factor for the development of pressure overload-induced LV hypertrophy [71, 79]. Elevated oxidative stress by exogenous H_2_O_2_ has also been reported to induce DNA damage (γH2A.X formation) in neonatal cardiomyocytes, as a consequence of ATM kinase activation [83]. It was, therefore, important to investigate whether the type 2A phosphatase-alpha4 signalling axis regulates the formation of oxidant stress-induced γH2A.X foci in cardiomyocytes. We have shown that all three type 2A phosphatase catalytic subunits are expressed in H9c2 cardiomyocytes and they have all been shown to dephosphorylate γH2A.X foci in non-myocytes [13, 67, 84]. However, the possibility of redundant phosphatase capacity within this system was addressed by knocking down the expression of just one of the catalytic subunits (PP6C) by >90% (Fig. 8a). Cardiomyocytes were then subjected to elevated levels of cellular oxidant stress (exogenous H_2_O_2_) at a concentration (300 μM) that induced pro-apoptotic signalling (Supplemental Figure S8) and increased the formation of γH2A.X foci in control-treated cells (3.4-fold). The formation of H_2_O_2_-induced γH2A.X foci (Fig. 8b) and expression of non-phosphorylated H2A.X protein (Fig. 8c) were not significantly altered by the knockdown of PP6C expression. However, when expression of alpha4 protein (>95%) was knocked down (Fig. 8d), the formation of γH2A.X foci in cardiomyocytes in response to H_2_O_2_ was completely abolished (Fig. 8e), which may have been dependent upon the significant reduction of total non-phosphorylated H2A.X protein in response to the knockdown of alpha4 protein expression (Fig. 8f). The phosphorylation status of H2A.X was also normalised to non-phosphorylated H2A.X (Supplemental Figure S9). It is worth noting that elevated levels of oxidant stress could differentially affect the expression of type 2A protein phosphatase catalytic subunits and alpha4 as determined by western analysis. The protein expression of both PP4C and PP6C was significantly (*P* < 0.05) reduced by exposure of cardiomyocytes to H_2_O_2_ (300 μM); however, the expression of PP2AC and alpha4 protein was unaltered (Supplemental Figure S10). Interestingly, PP6C has been shown to regulate levels of γH2A.X in non-myocytes; however, the knockdown of PP6C in cardiomyocytes was unable to significantly alter the formation of γH2A.X foci in the absence or presence of H_2_O_2_, thereby suggesting that the formation of γH2A.X in response to H_2_O_2_ does not involve PP6C, but is highly dependent on alpha4 protein expression. Furthermore, the expression of H2A.X histone protein was highly dependent upon the expression of alpha4 and not PP6C.

## Discussion

The original findings of the present study are that rat cardiomyocytes (embryonic, neonatal, and adult) express all type 2A protein phosphatases (PP2AC, PP4C and PP6C) at the mRNA level. We show that ARVM have relatively low levels of PP4C mRNA expression and undetectable levels of PP4C protein expression as determined by standard western analysis. The protein expression of all type 2A protein phosphatase catalytic subunits is differentially regulated by the 26S proteasome in ARVM and our data confirm that expression of the alpha4 protein is fundamentally important in maintaining type 2A protein phosphatase catalytic subunit protein expression in cardiomyocytes. Furthermore, this study also demonstrates that LV hypertrophy is not only associated with increased expression of PP2AC, but also alpha4, which we believe to be inextricably linked. Despite expression levels of PP6C protein being unaltered in hypertrophied tissue, our data show that LV hypertrophied tissue is associated with (1) increased expression of SAP1, (2) decreased expression of SAP2, and (3) increased expression of ANKRD28/44 PP6C regulatory proteins. Importantly, we also show that the cellular association of PP2AC and PP6C subunits with alpha4 protein was either unchanged or significantly decreased in the hypertrophied myocardium, respectively. The phosphorylation of phospholemman at Ser63 and Ser68 was dependent upon PP2ACα/β and PP4C, but not PP6C. Furthermore, the phosphorylation of a core histone (H2A) variant H2A.X on Ser139 (γH2A.X), a hallmark of DNA damage, was increased in response to oxidant stress (exogenous H_2_O_2_) in a PP6C-independent and alpha4-dependent manner.

This study describes for the first time the comparative expression levels (mRNA and protein) of all type 2A protein phosphatase catalytic subunits (PP2ACα/β, PP4C, and PP6C) in cardiomyocytes. We show that the mRNA encoding all four catalytic subunits is present in H9c2 cardiomyocytes, NRVMs, and ARVMs. The expression of PP2ACβ mRNA appears to be most abundant in embryonic H9c2 cardiomyocytes and NRVMs, whereas PP2ACβ mRNA in ARVM was significantly lower than PP2ACα mRNA, which suggests that PP2ACβ gene transcription is developmentally regulated in cardiomyocytes. In our study, levels of PP4C mRNA was significantly higher than PP6C mRNA in H9c2 rat cardiomyocytes, equivalent in NRVMs, but significantly lower than in ARVMs. This suggests that expression of the PP4C gene is developmentally regulated in the myocardium as in other tissues [32]. Our data suggest that type 2A catalytic subunit gene expression is qualitatively similar in H9c2 cardiomyocytes and NRVMs. As these cells come from different strains of the same species (rat), the data suggest that there are no strain-dependent differences in gene expression. In addition, despite PP2AC, PP4C and PP6C protein expression being present in both H9c2 cardiomyocytes (Fig. 1d) and NRVM (Supplemental Figure S1), PP4C protein was consistently absent in adult heart tissue (rat or mouse), which is supported by observations made by Kloeker et al. [39]. Furthermore, we were only able to observe PP4C protein expression in ARVM upon inhibition of the 26S proteasome with MG132, thereby suggesting that levels of PP4C protein expression in adult tissue were both transcriptionally (low mRNA) and post-translationally regulated. We believe that our data reveal an additional developmental difference relevant to the expression of PP4C protein, which may involve proteasomal targeting and degradation of PP4C in the adult myocardium. PP6C mRNA appears to be expressed to a similar degree in both ARVM and H9c2 cardiomyocytes; however, PP6C protein expression is significantly higher in ARVM than in H9c2 cardiomyocytes. This is suggestive that PP6C protein may be post-transcriptionally regulated or its stability altered in the adult myocardium. It is worth noting, however, that mild (1 μM) non-toxic inhibition of the 26S proteasome by MG132 did not significantly alter PP6C protein expression.

Several studies have shown that the non-catalytic regulatory protein alpha4 associates with type 2A catalytic subunits [11, 50, 54, 56]. In non-myocytes, the ablation of alpha4 protein expression has been reported to result in the indirect knockdown of PP2AC, PP4C, and PP6C protein expression [41, 44], thereby suggesting that cellular expression of the type 2A catalytic subunits is highly dependent on expression of alpha4 in cells. This indirect knockdown of type 2A catalytic subunits is underpinned by reports confirming that alpha4 contains an ubiquitin-interacting motif (UIM) that is thought to “protect” PP2AC from proteasome-mediated degradation, by diverting E3 ligase-mediated ubiquitination away from PP2AC [45, 49, 50]. This “protective” function of alpha4 protein towards PP2AC was confirmed to exist in cardiomyocytes by our data in Fig. 3, whereby the knockdown of alpha4 protein expression by siRNA also significantly attenuated the expression of type 2A catalytic subunits (PP2AC, PP4C, and PP6C). The significant development of LV hypertrophy in response to pressure overload as shown in Fig. 4a and Supplemental Figure S6, was sufficient to significantly increase PP2AC subunit expression by 65.2% in our studies, which supports the observations of previous reports that the diseased myocardium is causally associated with enhanced PP2AC expression [18, 25] and activity [1, 7]. It is worth noting, that in a similar model of pressure overload where transaortic constriction was also maintained for 4 weeks, the protein expression of a non-type 2A protein phosphatase, the dual-specificity phosphatase 14 (Dusp14) was conversely reduced in hypertrophied tissue [46]. Furthermore, rapid pacing has been shown to also decrease the protein expression of another dual-specificity phosphatase MAPK phosphatase 1 (MKP1) [22]. This suggests that the expression of functionally distinct phosphatase families may be differentially altered in the diseased myocardium. However, the nature of the elevated PP2AC protein expression in the diseased myocardium is poorly understood. Gergs et al. [25] reported that the overexpression of PP2AC in the murine myocardium induced LV hypertrophy but without any change in the expression of alpha4, thereby suggesting that PP2AC protein expression per se does not regulate expression of alpha4 protein. However, in our study, expression of alpha4 protein was significantly increased in hypertrophied tissue and a significance of this increase may explain why there was a concomitant significant increase in PP2AC in hypertrophied tissue. Hence, our study suggests that the pressure overload-induced increase in alpha4 protein expression may have protected PP2AC from degradation by the 26S proteasome (discussed earlier) and consequently augmented the amount of PP2AC protein in hypertrophied tissue as seen in other studies [18, 25]. It is worth noting that in non-myocytes, alpha4 overexpression can significantly increase ectopic PP2AC overexpression [41, 45, 82]; however, overexpression of alpha4 per se does not affect native levels of PP2AC expression [41, 50]. We propose that there is an increase in PP2AC:alpha4 complex formation in cardiac tissue subjected to pressure overload. Interestingly, alpha4 protein expression has been found to be elevated in other non-cardiac tissues undergoing uncontrolled growth [12, 69]. However, an explanation of why alpha4 protein expression was elevated in hypertrophied tissue remains an important question that requires further study.

The role(s) of the other type 2A phosphatase catalytic subunits (PP4C and PP6C) in cardiovascular pathologies is currently undefined. The absence of PP4C protein expression in the adult myocardium precluded us from studying the expression of PP4C regulatory proteins in the heart and our data show that protein expression of the PP6C subunit is not altered by pressure overload-induced hypertrophy. Hence, we focussed on the subunits that are thought to regulate targeting and activity of PP6C, which as our data suggest is abundantly expressed in the adult myocardium. Evidence suggests that the sit4-associated proteins, SAP1 and SAP2, regulate the phosphorylation of H2A.X in either positive or negative manners, respectively. Studies have shown that SAP1/PP6C protein complexes associate with, dephosphorylate and activate DNA-PK [31, 51], which then phosphorylates the nuclear histone (H2A) variant H2A.X at Ser139 (γH2A.X) [8]. On the other hand, SAP2/PP6C protein has been shown to dephosphorylate γH2A.X following DNA damage [84]. In our studies, novel data suggest that even though PP6C protein expression was unaltered in hypertrophied tissue, protein expression of PP6C regulatory proteins SAP1 and SAP2 was either increased or reduced, respectively. This would argue for increased levels of γH2A.X in hypertrophied tissue, which, in our studies, was not the case (Supplemental Figure S5).

To date, the role(s) of ANKRD28/44/52 proteins in both cardiac health and disease are undefined. This is the first study to report the existence of the PP6C regulatory ANKRD proteins in the heart, and it is, therefore, difficult to ascribe any consequence of the elevated ANKRD28 or ANKRD44 protein expression observed in the hypertrophied myocardium. Seeing as ANKRD proteins bind SAPs which themselves are associated with PP6C subunits [77], one can only infer that the increased expression of ANKRD28 and ANKRD42 proteins may mediate increased localisation/activity of PP6C within the hypertrophied myocardium. In our study, elevated ANKRD28/44 protein expression which would promote γH2A.X dephosphorylation may override the effects of altered SAP1 & SAP2 expression which might promote H2A.X phosphorylation, thereby contributing to the lower, albeit non-significant, levels of γH2A.X in hypertrophied tissue (Supplemental Figure S5).

The association between alpha4 protein and PP6C is known to inhibit PP6C activity in vitro [56, 63] and in yeast [35]. Recent evidence suggests that a significantly higher proportion of cellular PP6C, compared to PP2AC or PP4C, is found associated with alpha4 protein in HEK293T cells [44]. These data partly agree with what we have observed, except that alpha4 protein also associates with a significant proportion of PP2AC in the adult myocardium. Furthermore, our study describes novel observations regarding the association between alpha4 and type 2A catalytic subunits in diseased tissue. Although the association between alpha4 and PP2AC was unaltered, despite an increase in the expression of both proteins in hypertrophied myocardium, the association between alpha4 and PP6C was significantly reduced. A decreased association between alpha4 and PP6C protein would be expected to perhaps disinhibit PP6C, thereby leading to enhanced PP6C activity. Hence, disinhibited PP6C combined with elevated levels of ANKRD28/44 protein expression in hypertrophied tissue may contribute to the dephosphorylation of substrates such as H2A.X in hypertrophied tissue (Supplemental Figure S5).

PP2AC has been implicated as a protein phosphatase that regulates the activity of many ion transporters involved in cardiomyocyte excitation–contraction coupling. One such ion transporter is the sodium pump, whose activity is regulated through its association with the phosphoprotein PLM [17]. The phosphorylation of PLM on Ser63 and Ser68 relieves its inhibitory activity towards the sodium pump and thereby accelerates the efflux of sodium ions from the cardiomyocyte (see review by Pavlovic et al. [62]). Hence, hypophosphorylation of PLM at Ser63 and ser68 would be expected to decrease sodium pump activity, reduce sodium efflux, and as a consequence augment intracellular sodium load within the cardiomyocyte. In hypertrophied left ventricular tissue, Boguslavskyi et al. reported that the phosphorylation of PLM at both Ser63 and Ser68 was significantly reduced, thereby contributing to elevated intracellular sodium levels [6]. Our own data (Fig. 7) suggest that not only PP2ACα/β, but also PP4C can induce the dephosphorylation of PLM at Ser63 and Ser68 in cardiomyocytes. In the context of myocardial hypertrophy, as PP4C is not expressed in adult myocardial tissue, it is, therefore, unlikely to contribute to regulating the phosphorylation status of PLM in adult hypertrophied LV tissue. Furthermore, despite having shown that the expression of PP6C regulatory proteins (ANKRD28/44) is elevated in LV hypertrophied tissue and our catalytic subunit knockdown data suggest that PP6C does not dephosphorylate PLM, it is unlikely that PP6C plays any significant role in regulating the phosphorylation status of Ser63 and Ser68 of PLM in hypertrophied LV myocardium. It is, therefore, very likely that the only type 2A protein phosphatase isoforms that regulate the phosphorylation of PLM in the healthy or hypertrophied heart are PP2AC α and β.

The phosphorylation of H2A.X at Ser139 and formation of γH2A.X foci are considered a hallmark of DNA damage in cells [48]. Oxidant stress is a potent inducer of DNA damage and consequent formation of γH2A.X foci in non-myocytes [38, 66]. Furthermore, H_2_O_2_ induces DNA damage (γH2A.X formation) in neonatal cardiomyocytes, as a consequence of ATM kinase activation [83]. In support, our data show that cellular oxidant stress (300 μM H_2_O_2_) not only increased levels of γH2A.X foci, but also induced apoptotic signalling in cardiomyocytes by promoting the cleavage and activation of (pro)caspase 3 and inactivation of a caspase 3 substrate PARP1 (Supplemental Figure S8). Despite PP6C being implicated in regulating the formation of γH2A.X foci in non-myocytes [21, 84], the knockdown of PP6C protein expression in cardiomyocytes did not increase the basal formation of γH2A.X foci or augment those formed in response to H_2_O_2_. This suggests that either PP6C does not target γH2A.X in cardiomyocytes or that there is sufficient redundant activity of other type 2A catalytic subunits (PP2AC and PP4C) towards γH2A.X to compensate for the siRNA-induced knockdown of PP6C. Our data suggest that PP2AC may be the probable candidate as its expression is unaltered by H_2_O_2_, whereas PP4C expression was significantly attenuated by exposure to H_2_O_2_ (Supplemental Figure S10). The differential effect of oxidant stress on type 2A catalytic subunits is supported (in part) by a lack of effect of H_2_O_2_ on alpha4 expression. The knockdown of alpha4 expression was not only shown to attenuate the expression of all type 2A catalytic subunits but to significantly decrease the formation of γH2A.X foci in untreated cardiomyocytes and those exposed to H_2_O_2_. This was unexpected as reports have indicated that all type 2A catalytic subunits are known to target γH2A.X [13, 21, 67], and indirect knockdown of these catalytic subunits by silencing the expression of alpha4 protein should have increased levels of γH2A.X as reported by Kong et al. [41]. Interestingly, core histones (H2A) in cells exposed to significant stress and consequent DNA damage can be degraded by the PA200/Bim10-containing proteasome [65], so that damaged DNA can be unravelled and repaired by the DNA damage repair response. We propose that, in our study, the alpha4 knockdown and consequent loss of type 2A catalytic subunits constituted a severe insult. We propose that the severity of this insult was sufficient to induce the degradation of H2A.X protein and prevent cells from forming γH2A.X foci, thereby failing to undergo DNA repair to maintain genomic stability and cellular integrity. Hypertrophic tissue might be expected to exhibit elevated levels of γH2A.X foci, as it is associated with higher levels of oxidative stress [71, 79]. However, our data suggest that this is not the case and that γH2A.X foci levels were if anything slightly lower in hypertrophied tissue. Maintaining low levels of γH2A.X foci may well delay DNA repair in myocytes subjected to a higher degree of cell stress and may, therefore, predispose these myocytes to eventual cell injury and death.

In conclusion, these studies indicate that the type 2A protein phosphatases are differentially regulated in both the healthy and hypertrophied myocardium. The data suggest that pressure overload-induced hypertrophy is associated with (1) altered expression of type 2A protein phosphatases and their regulatory subunits and (2) an increase in expression of their non-catalytic regulatory protein alpha4. Interestingly, PP6C was shown to associate with alpha4 protein in adult cardiac tissue as in other cell types, but its association with alpha4 protein was significantly reduced in hypertrophied tissue. In addition, despite elevated expression of both PP2AC and alpha4 protein, the association between PP2AC with alpha4 protein was unaltered in hypertrophied tissue. The phosphorylation status of PLM at Ser63 and Ser68 is regulated by PP2ACα, PP2ACβ, and PP4C. The expression of the core histone H2A.X and formation of γH2A.X foci in response to H_2_O_2_ are not regulated by PP6C, but is highly dependent on alpha4 protein expression in cardiomyocytes.

## Electronic supplementary material

Below is the link to the electronic supplementary material.

Supplementary material 2 (PPTX 1057 kb)
