# Supplementary material for: Expression and regulation of type 2A protein phosphatases and alpha4 signalling in cardiac health and hypertrophy
Source: Basic Res Cardiol. 2017 May 19;112(4):37. doi: 10.1007/s00395-017-0625-2 (PMC5438423; doi:10.1007/s00395-017-0625-2)
Supplement: Supplementary file 1 — Supplementary material 2 (PPTX 1057 kb) [file 395_2017_625_MOESM1_ESM.pptx]

## Slide 1
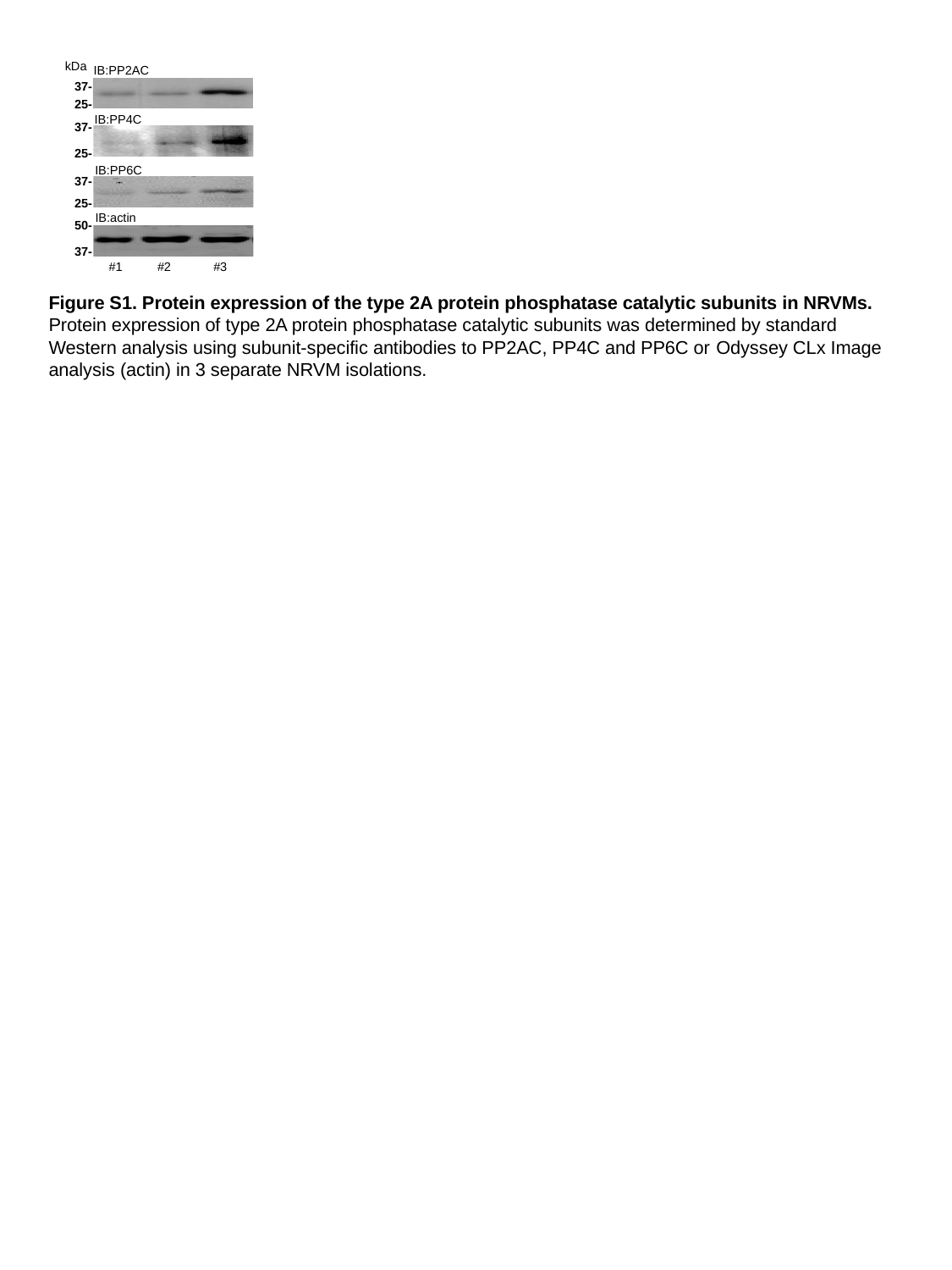

kDa
IB:PP2AC
37-
25-
IB:PP4C
37-
25-
IB:PP6C
37-
25-
IB:actin
50-
37-
#1
#2
#3
Figure S1. Protein expression of the type 2A protein phosphatase catalytic subunits in NRVMs. Protein expression of type 2A protein phosphatase catalytic subunits was determined by standard Western analysis using subunit-specific antibodies to PP2AC, PP4C and PP6C or Odyssey CLx Image analysis (actin) in 3 separate NRVM isolations.

## Slide 2
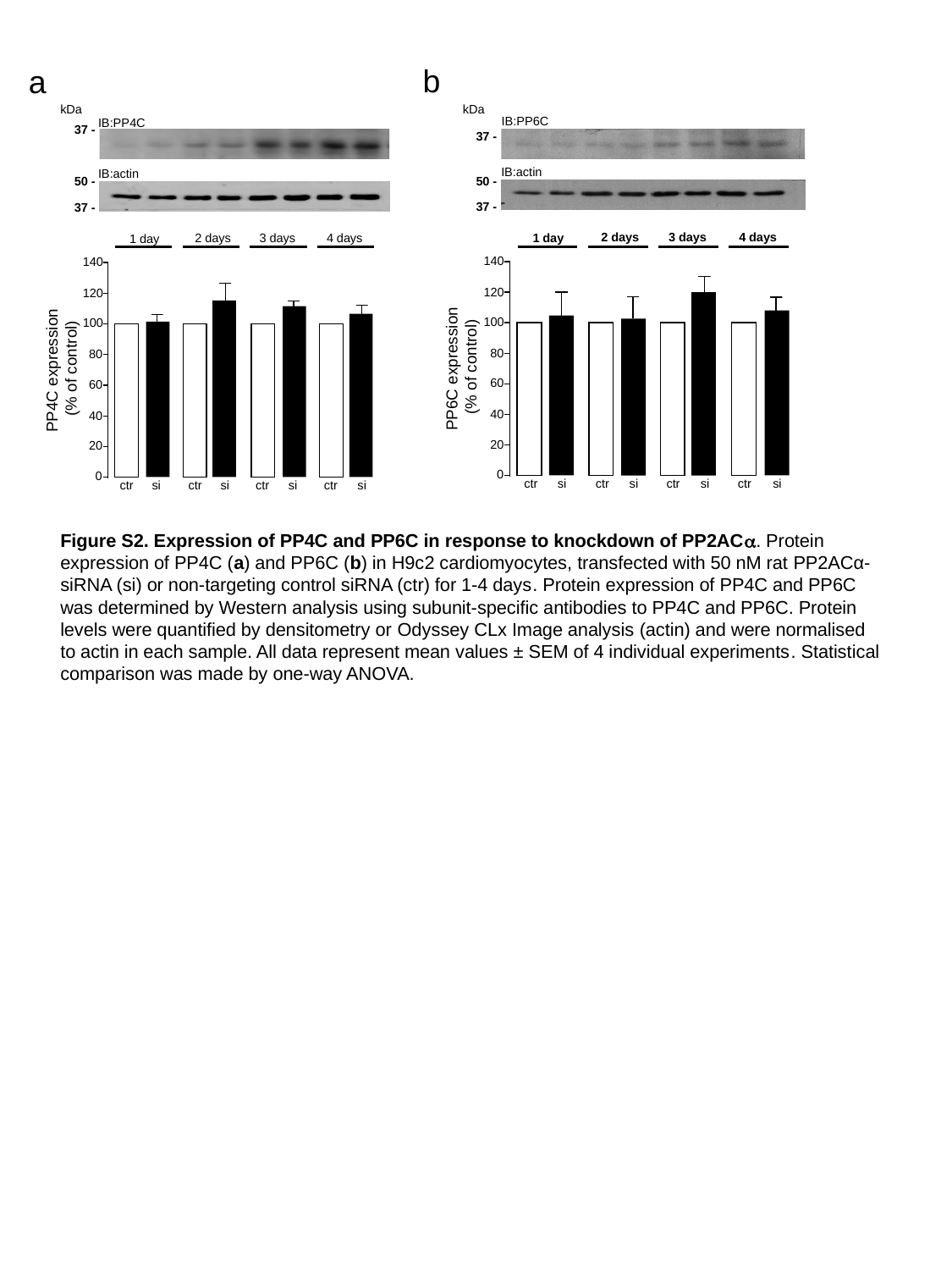

b
a
kDa
kDa
IB:PP6C
IB:PP4C
37 -
37 -
IB:actin
IB:actin
50 -
50 -
37 -
37 -
2 days
3 days
4 days
1 day
2 days
3 days
4 days
1 day
140
140
120
120
100
100
PP6C expression
(% of control)
PP4C expression
(% of control)
80
80
60
60
40
40
20
20
0
0
ctr
si
ctr
si
ctr
si
ctr
si
ctr
si
ctr
si
ctr
si
ctr
si
Figure S2. Expression of PP4C and PP6C in response to knockdown of PP2ACa. Protein expression of PP4C (a) and PP6C (b) in H9c2 cardiomyocytes, transfected with 50 nM rat PP2ACα-siRNA (si) or non-targeting control siRNA (ctr) for 1-4 days. Protein expression of PP4C and PP6C was determined by Western analysis using subunit-specific antibodies to PP4C and PP6C. Protein levels were quantified by densitometry or Odyssey CLx Image analysis (actin) and were normalised to actin in each sample. All data represent mean values ± SEM of 4 individual experiments. Statistical comparison was made by one-way ANOVA.

## Slide 3
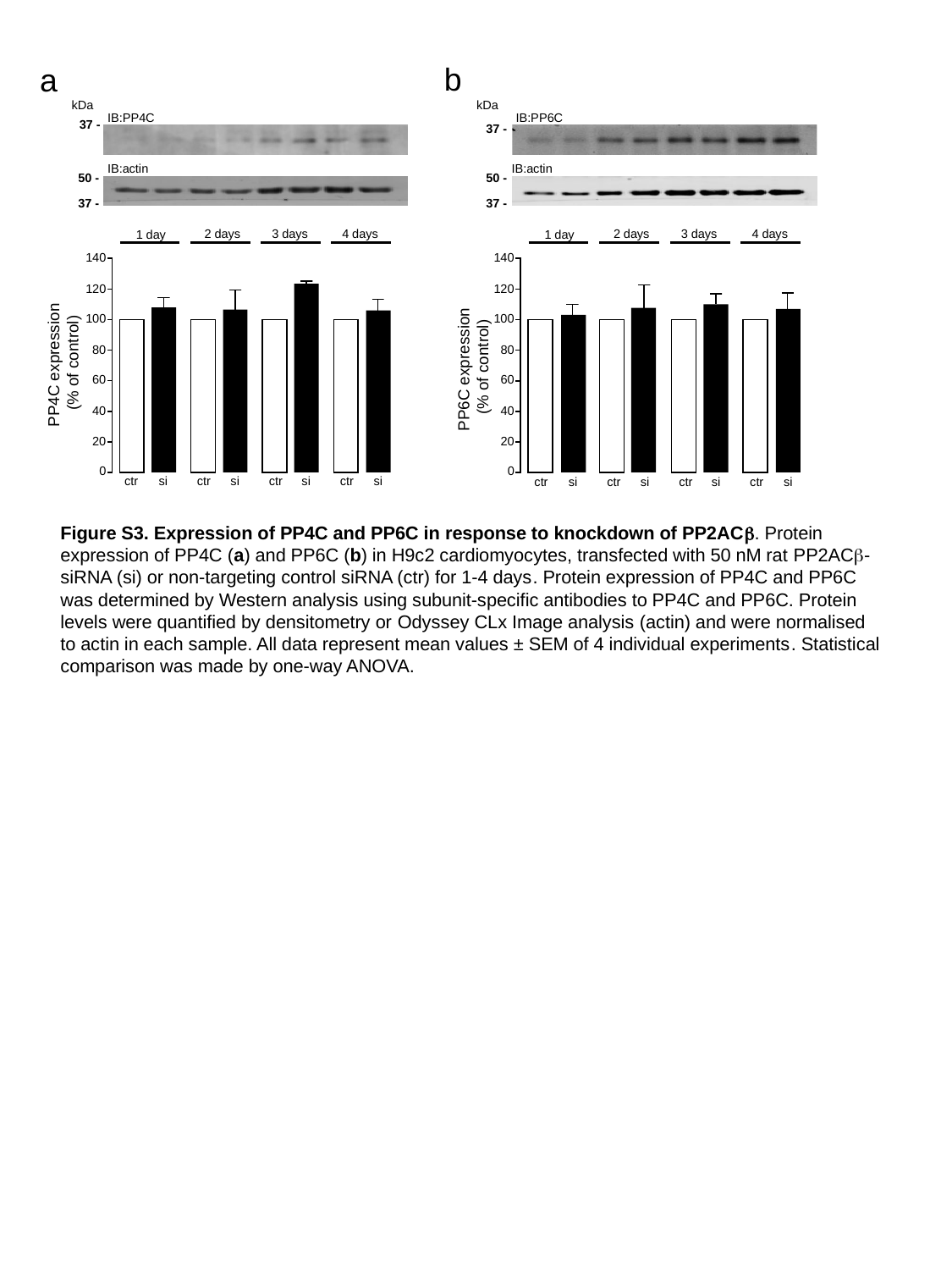

b
a
kDa
kDa
IB:PP4C
IB:PP6C
37 -
37 -
IB:actin
IB:actin
50 -
50 -
37 -
37 -
2 days
3 days
4 days
2 days
3 days
4 days
1 day
1 day
140
140
120
120
100
100
PP4C expression
(% of control)
80
80
PP6C expression
(% of control)
60
60
40
40
20
20
0
0
ctr
si
ctr
si
ctr
si
ctr
si
ctr
si
ctr
si
ctr
si
ctr
si
Figure S3. Expression of PP4C and PP6C in response to knockdown of PP2ACb. Protein expression of PP4C (a) and PP6C (b) in H9c2 cardiomyocytes, transfected with 50 nM rat PP2ACβ-siRNA (si) or non-targeting control siRNA (ctr) for 1-4 days. Protein expression of PP4C and PP6C was determined by Western analysis using subunit-specific antibodies to PP4C and PP6C. Protein levels were quantified by densitometry or Odyssey CLx Image analysis (actin) and were normalised to actin in each sample. All data represent mean values ± SEM of 4 individual experiments. Statistical comparison was made by one-way ANOVA.

## Slide 4
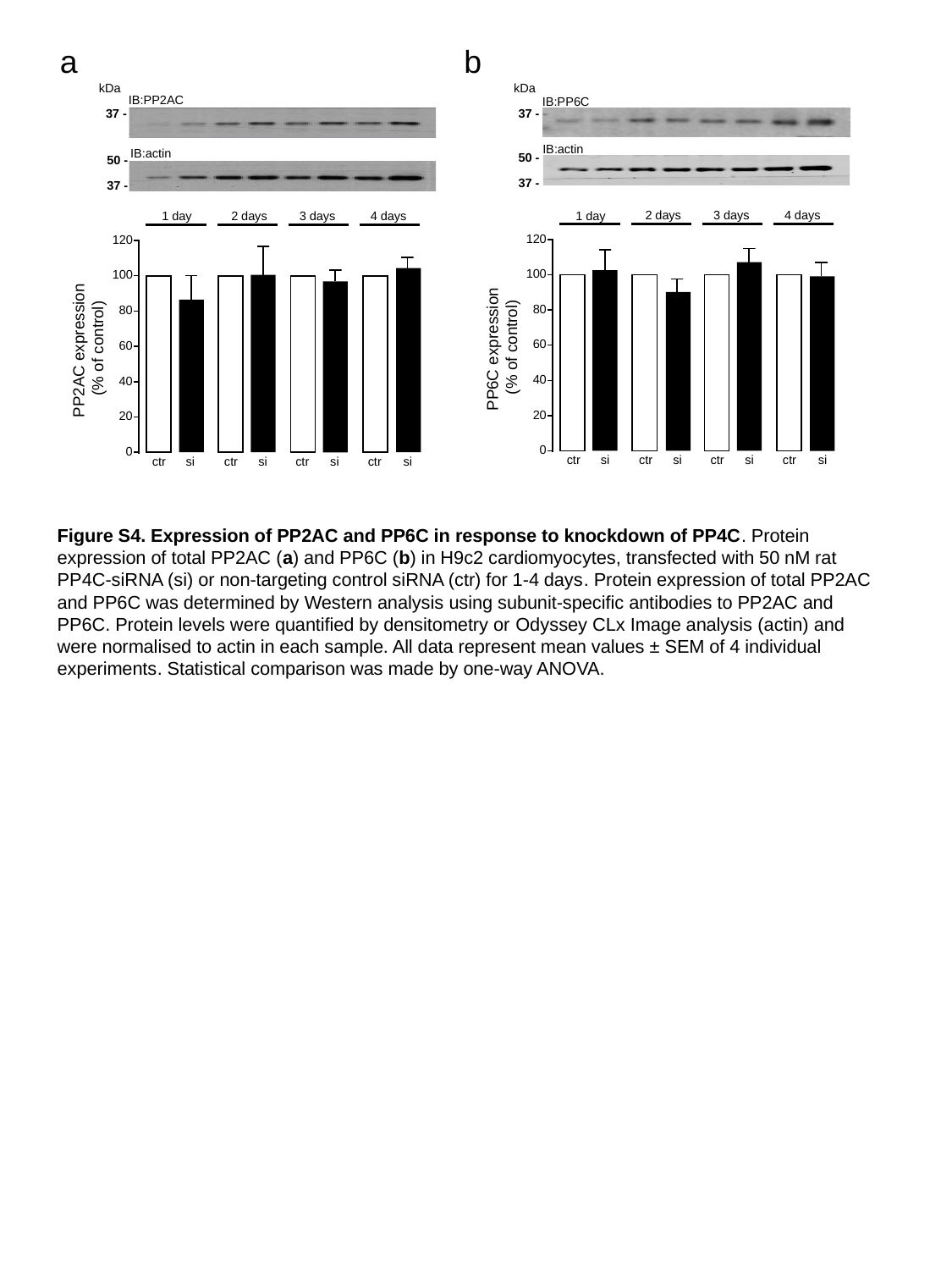

b
a
kDa
kDa
IB:PP2AC
IB:PP6C
37 -
37 -
IB:actin
IB:actin
50 -
50 -
37 -
37 -
2 days
3 days
4 days
2 days
3 days
4 days
1 day
1 day
120
120
100
100
80
80
PP6C expression
(% of control)
PP2AC expression
(% of control)
60
60
40
40
20
20
0
0
ctr
si
ctr
si
ctr
si
ctr
si
ctr
si
ctr
si
ctr
si
ctr
si
Figure S4. Expression of PP2AC and PP6C in response to knockdown of PP4C. Protein expression of total PP2AC (a) and PP6C (b) in H9c2 cardiomyocytes, transfected with 50 nM rat PP4C-siRNA (si) or non-targeting control siRNA (ctr) for 1-4 days. Protein expression of total PP2AC and PP6C was determined by Western analysis using subunit-specific antibodies to PP2AC and PP6C. Protein levels were quantified by densitometry or Odyssey CLx Image analysis (actin) and were normalised to actin in each sample. All data represent mean values ± SEM of 4 individual experiments. Statistical comparison was made by one-way ANOVA.

## Slide 5
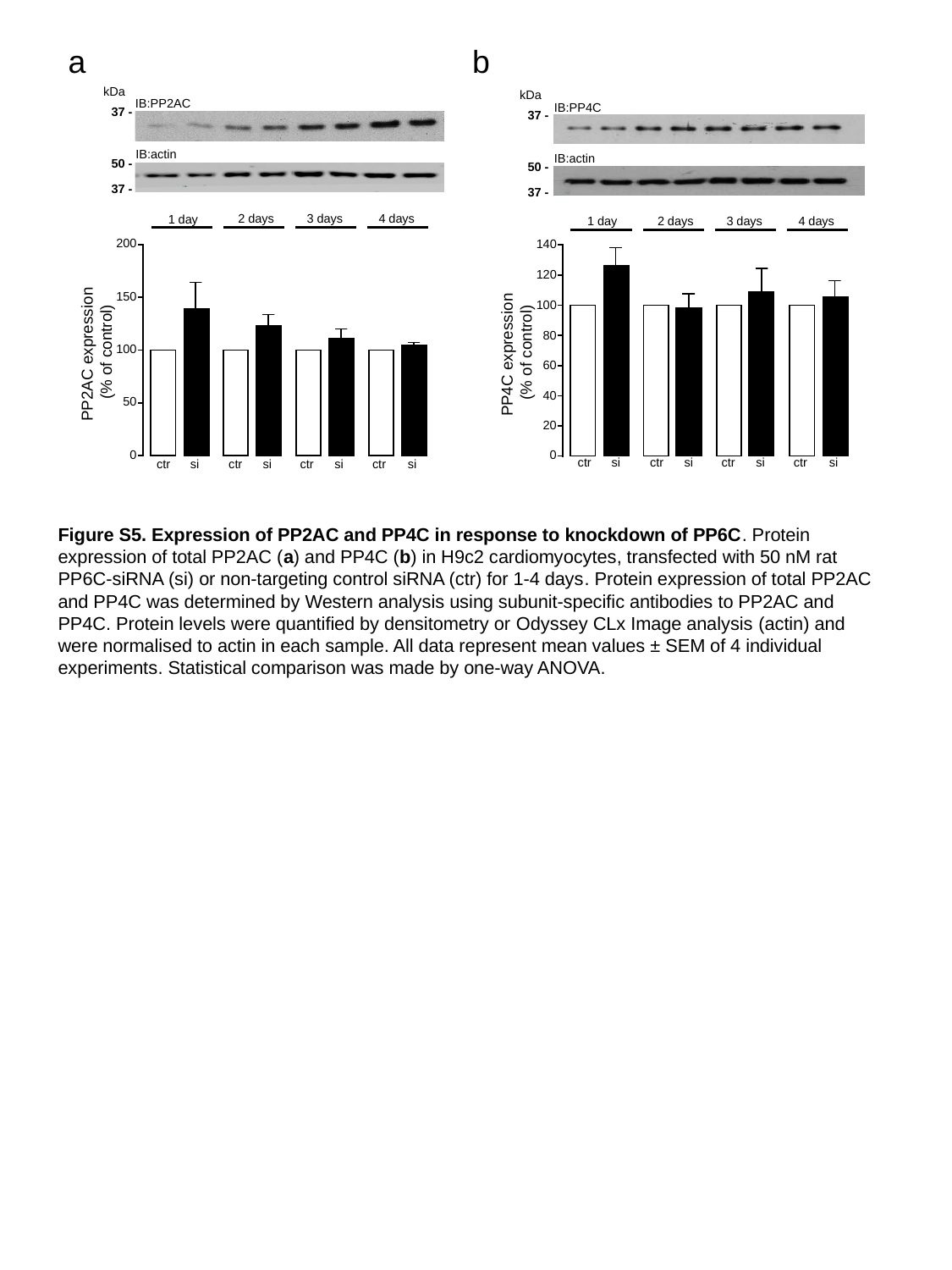

a
b
kDa
kDa
IB:PP2AC
IB:PP4C
37 -
37 -
IB:actin
IB:actin
50 -
50 -
37 -
37 -
2 days
3 days
4 days
1 day
2 days
3 days
4 days
1 day
200
140
120
150
100
PP2AC expression
(% of control)
PP4C expression
(% of control)
80
100
60
40
50
20
0
0
ctr
si
ctr
si
ctr
si
ctr
si
ctr
si
ctr
si
ctr
si
ctr
si
Figure S5. Expression of PP2AC and PP4C in response to knockdown of PP6C. Protein expression of total PP2AC (a) and PP4C (b) in H9c2 cardiomyocytes, transfected with 50 nM rat PP6C-siRNA (si) or non-targeting control siRNA (ctr) for 1-4 days. Protein expression of total PP2AC and PP4C was determined by Western analysis using subunit-specific antibodies to PP2AC and PP4C. Protein levels were quantified by densitometry or Odyssey CLx Image analysis (actin) and were normalised to actin in each sample. All data represent mean values ± SEM of 4 individual experiments. Statistical comparison was made by one-way ANOVA.

## Slide 6
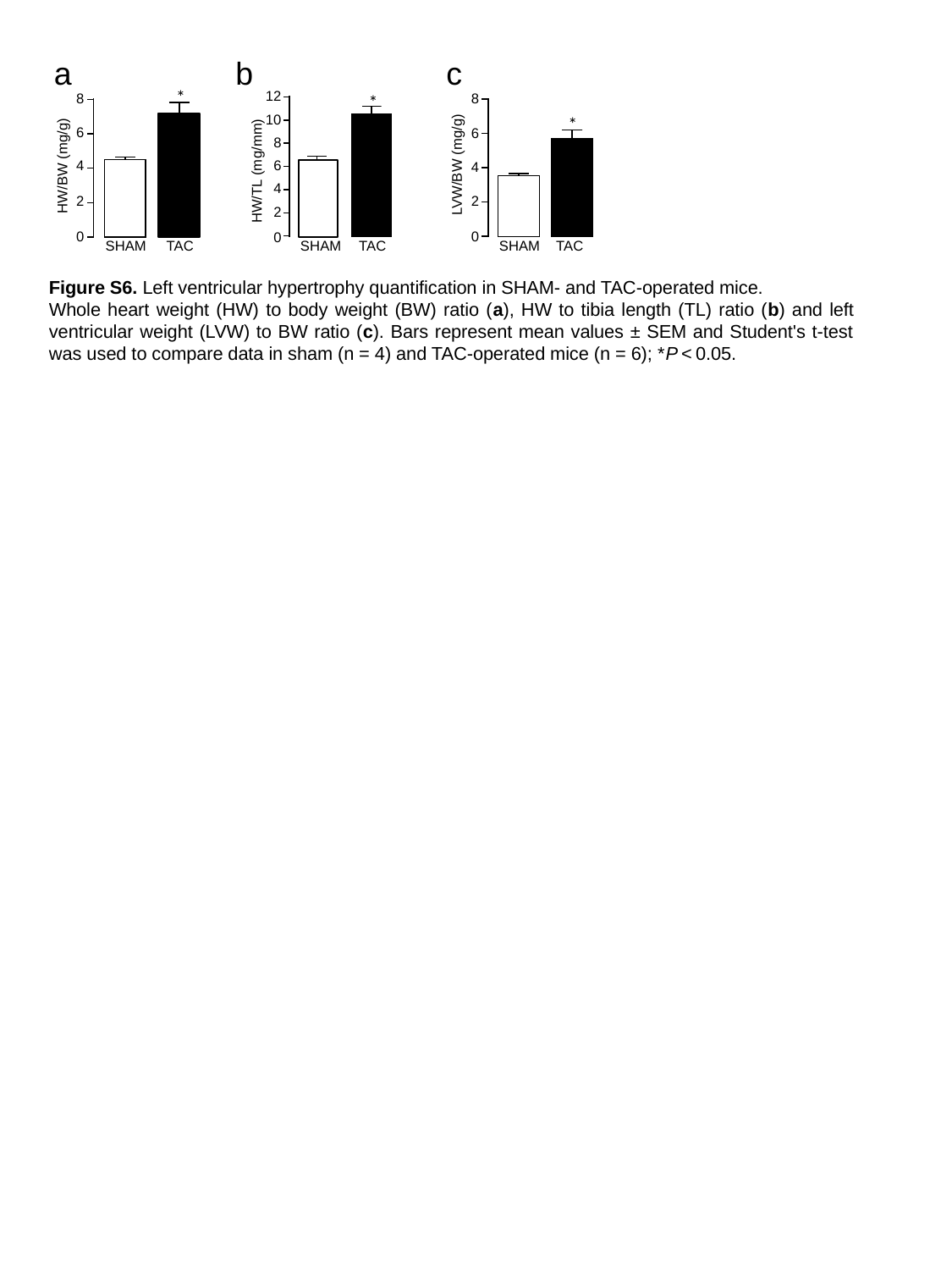

a
b
c
*
*
12
8
8
*
10
6
6
8
LVW/BW (mg/g)
HW/BW (mg/g)
HW/TL (mg/mm)
6
4
4
4
2
2
2
0
0
0
SHAM
TAC
SHAM
TAC
SHAM
TAC
Figure S6. Left ventricular hypertrophy quantification in SHAM- and TAC-operated mice.
Whole heart weight (HW) to body weight (BW) ratio (a), HW to tibia length (TL) ratio (b) and left ventricular weight (LVW) to BW ratio (c). Bars represent mean values ± SEM and Student's t-test was used to compare data in sham (n = 4) and TAC-operated mice (n = 6); *P < 0.05.

## Slide 7
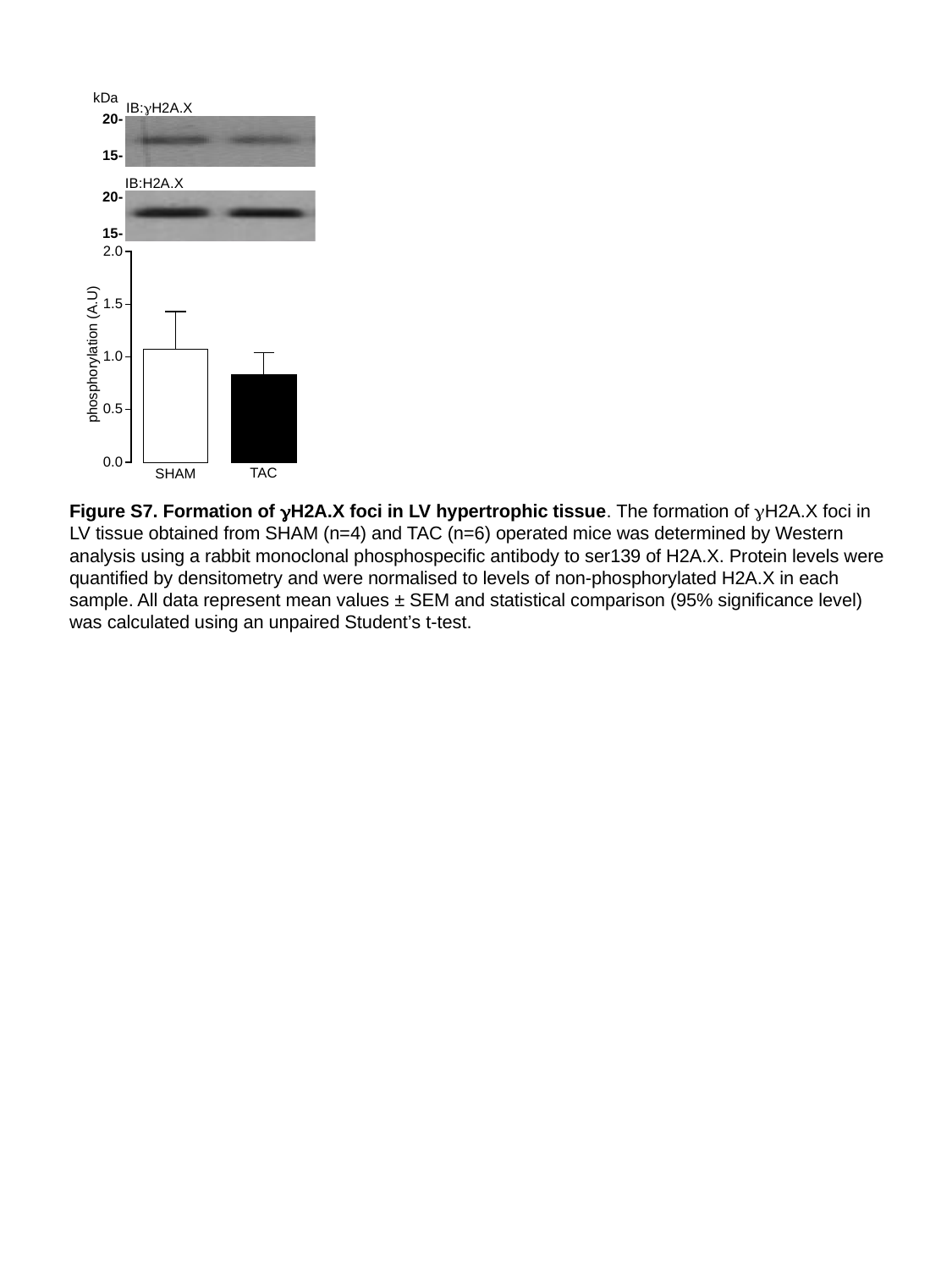

kDa
IB:gH2A.X
20-
15-
IB:H2A.X
20-
15-
2.0
1.5
phosphorylation (A.U)
1.0
0.5
0.0
TAC
SHAM
Figure S7. Formation of gH2A.X foci in LV hypertrophic tissue. The formation of gH2A.X foci in LV tissue obtained from SHAM (n=4) and TAC (n=6) operated mice was determined by Western analysis using a rabbit monoclonal phosphospecific antibody to ser139 of H2A.X. Protein levels were quantified by densitometry and were normalised to levels of non-phosphorylated H2A.X in each sample. All data represent mean values ± SEM and statistical comparison (95% significance level) was calculated using an unpaired Student’s t-test.

## Slide 8
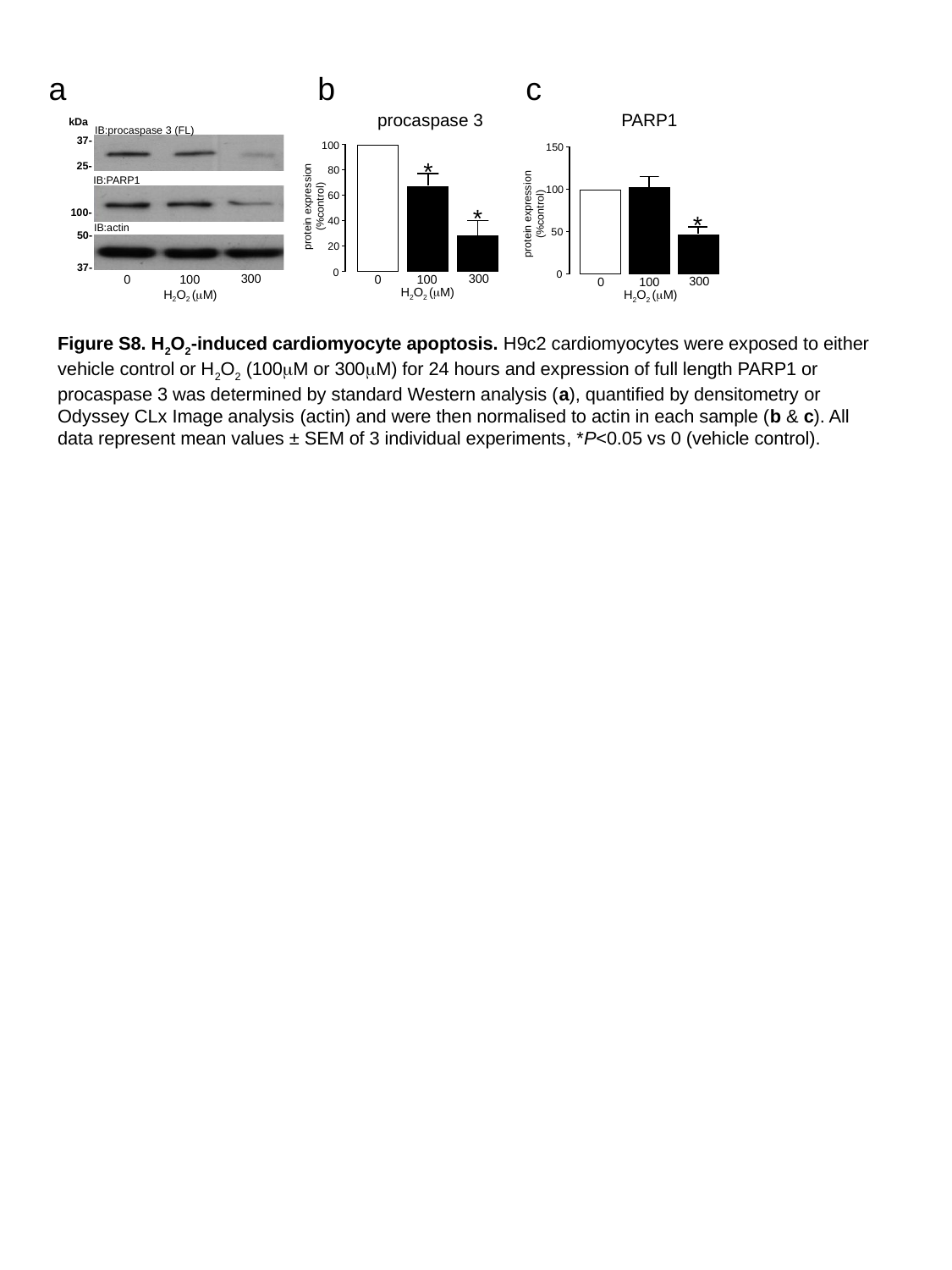

a
b
c
procaspase 3
PARP1
kDa
IB:procaspase 3 (FL)
37-
25-
100
150
*
80
IB:PARP1
100-
100
60
protein expression
(%control)
*
protein expression
(%control)
*
40
IB:actin
50-
50
20
37-
0
0
300
300
0
100
0
100
300
0
100
H2O2 (mM)
H2O2 (mM)
H2O2 (mM)
Figure S8. H2O2-induced cardiomyocyte apoptosis. H9c2 cardiomyocytes were exposed to either vehicle control or H2O2 (100mM or 300mM) for 24 hours and expression of full length PARP1 or procaspase 3 was determined by standard Western analysis (a), quantified by densitometry or Odyssey CLx Image analysis (actin) and were then normalised to actin in each sample (b & c). All data represent mean values ± SEM of 3 individual experiments, *P<0.05 vs 0 (vehicle control).

## Slide 9
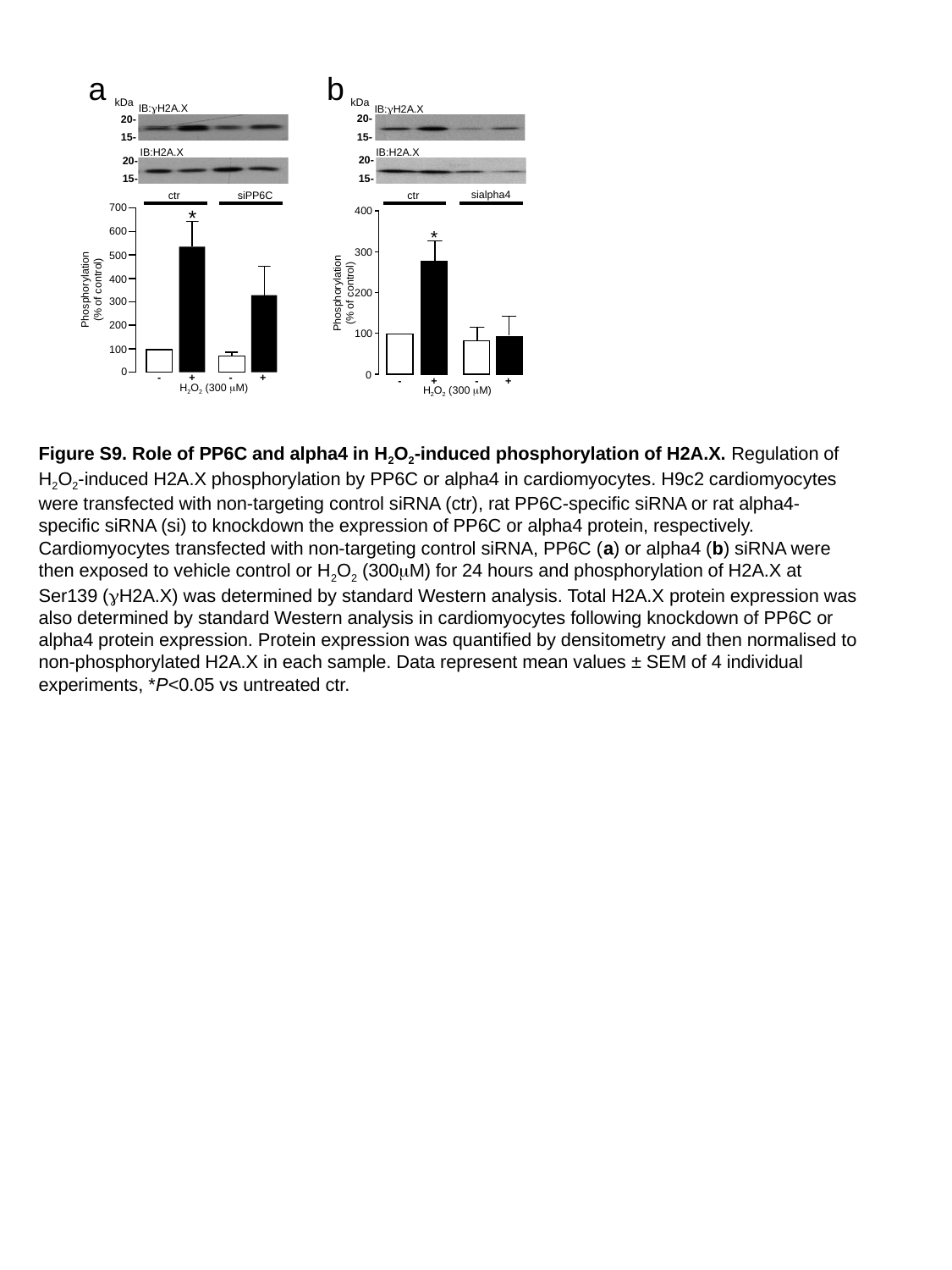

a
b
kDa
kDa
IB:gH2A.X
IB:gH2A.X
20-
20-
15-
15-
IB:H2A.X
IB:H2A.X
20-
20-
15-
15-
sialpha4
ctr
siPP6C
ctr
700
*
400
600
*
300
500
Phosphorylation
(% of control)
400
Phosphorylation
(% of control)
200
300
200
100
100
0
0
-
+
-
+
-
+
-
+
H2O2 (300 mM)
H2O2 (300 mM)
Figure S9. Role of PP6C and alpha4 in H2O2-induced phosphorylation of H2A.X. Regulation of H2O2-induced H2A.X phosphorylation by PP6C or alpha4 in cardiomyocytes. H9c2 cardiomyocytes were transfected with non-targeting control siRNA (ctr), rat PP6C-specific siRNA or rat alpha4-specific siRNA (si) to knockdown the expression of PP6C or alpha4 protein, respectively. Cardiomyocytes transfected with non-targeting control siRNA, PP6C (a) or alpha4 (b) siRNA were then exposed to vehicle control or H2O2 (300mM) for 24 hours and phosphorylation of H2A.X at Ser139 (gH2A.X) was determined by standard Western analysis. Total H2A.X protein expression was also determined by standard Western analysis in cardiomyocytes following knockdown of PP6C or alpha4 protein expression. Protein expression was quantified by densitometry and then normalised to non-phosphorylated H2A.X in each sample. Data represent mean values ± SEM of 4 individual experiments, *P<0.05 vs untreated ctr.

## Slide 10
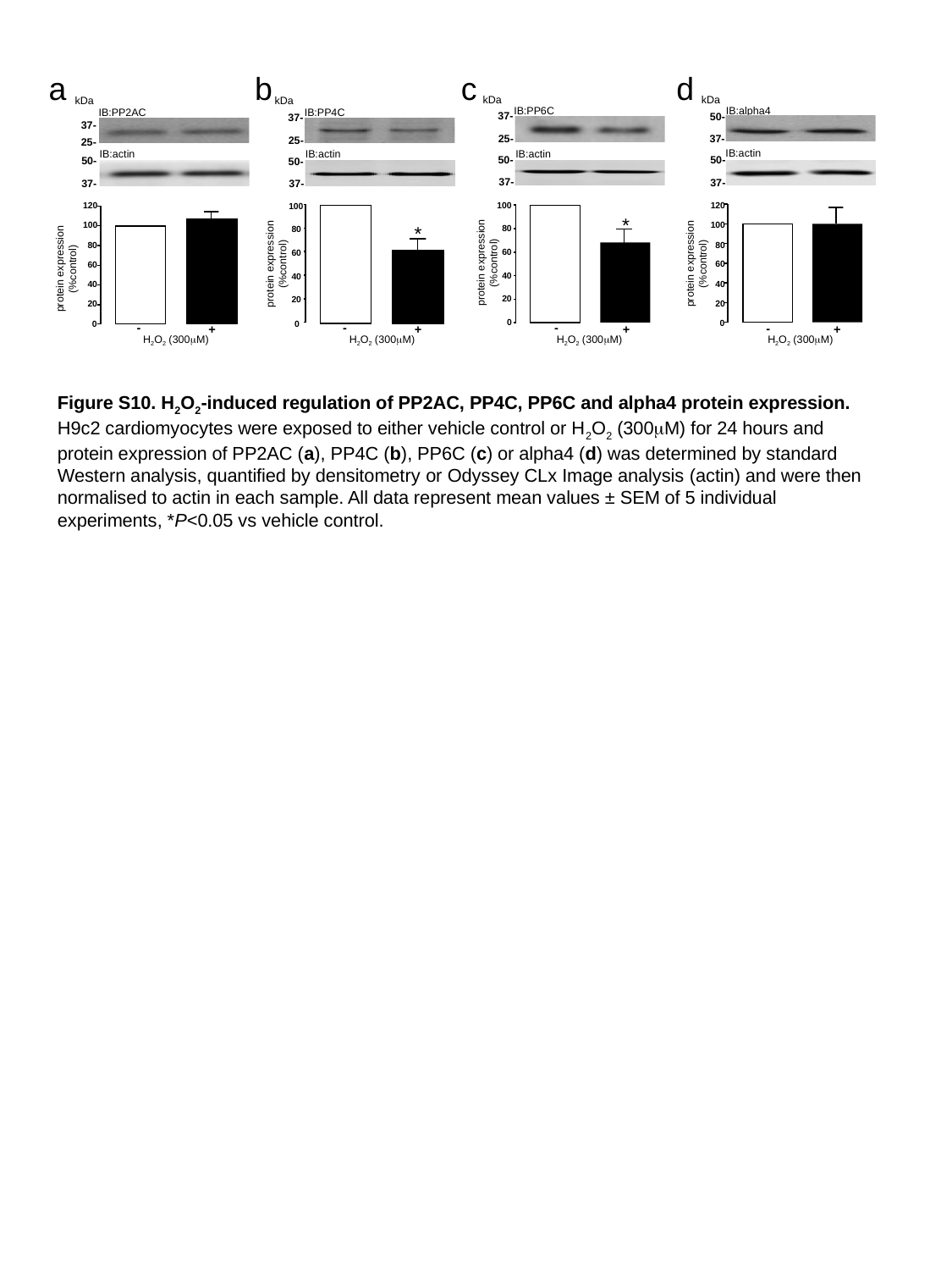

a
b
c
d
kDa
kDa
kDa
kDa
IB:alpha4
IB:PP6C
IB:PP2AC
IB:PP4C
37-
50-
37-
37-
25-
37-
25-
25-
IB:actin
IB:actin
IB:actin
IB:actin
50-
50-
50-
50-
37-
37-
37-
37-
100
120
120
100
80
60
40
20
0
100
*
100
*
80
80
80
60
60
protein expression
(%control)
protein expression
(%control)
protein expression
(%control)
protein expression
(%control)
60
40
40
40
20
20
20
0
0
0
-
-
-
-
+
+
+
+
H2O2 (300mM)
H2O2 (300mM)
H2O2 (300mM)
H2O2 (300mM)
Figure S10. H2O2-induced regulation of PP2AC, PP4C, PP6C and alpha4 protein expression. H9c2 cardiomyocytes were exposed to either vehicle control or H2O2 (300mM) for 24 hours and protein expression of PP2AC (a), PP4C (b), PP6C (c) or alpha4 (d) was determined by standard Western analysis, quantified by densitometry or Odyssey CLx Image analysis (actin) and were then normalised to actin in each sample. All data represent mean values ± SEM of 5 individual experiments, *P<0.05 vs vehicle control.
